# Association between body roundness index and hearing loss in the United States population: A cross-sectional study

**DOI:** 10.1097/MD.0000000000044401

**Published:** 2025-10-03

**Authors:** Guoxiong Li, Shangjin Yang, Yuzhou Cai, Shuang Liu, Haixia Wu, Chengmin Shi, Yujian Zeng

**Affiliations:** a Department of Gastrointestinal Surgery, The First Affiliated Hospital of Kunming Medical University, Kunming, Yunnan, China; b Department of Ultrasound, The First Affiliated Hospital of Kunming Medical University, Kunming, Yunnan, China; c Department of Breast Surgery, The First Affiliated Hospital of Kunming Medical University, Kunming, Yunnan, China.

**Keywords:** body roundness index, hearing loss, machine learning, NHANES, obesity

## Abstract

Hearing loss represents a significant public health concern, with obesity increasingly recognized as a potential risk factor. The body roundness index (BRI) is a novel anthropometric measure that more accurately reflects body fat distribution in comparison to conventional obesity indices. Nevertheless, the relationship between BRI and hearing loss remains to be elucidated. This cross-sectional study utilized data from the National Health and Nutrition Examination Survey to investigate the relationship between BRI and hearing loss in United States adults. Hearing loss was categorized into low frequency, high frequency, and speech-frequency categories based on audiometric testing. The association between BRI and hearing impairment was then assessed using a range of analytical approaches, including logistic regression models, restricted cubic spline analysis, and machine learning methods (least absolute shrinkage and selection operator, random forest, and eXtreme Gradient Boosting). Higher BRI was significantly associated with increased odds of hearing loss across all frequency domains, even after adjusting for potential confounders. A nonlinear relationship was observed, with a threshold effect indicating a stronger association at lower BRI levels. Machine learning models confirmed the predictive value of BRI, with shapley additive explanations analysis highlighting its importance relative to other metabolic and environmental factors. BRI has been demonstrated to be a significant predictor of hearing loss, thus suggesting that central obesity may contribute to auditory dysfunction. These findings emphasize the necessity for obesity management to be incorporated into hearing loss prevention strategies, and they underscore BRI as a potential screening tool for identifying individuals at risk of auditory impairment.

## 1. Introduction

Hearing loss represents a universal health concern, impacting individuals across diverse socioeconomic strata. According to the World Health Organization, approximately 1.5 billion people worldwide contend with varying degrees of hearing impairment, of whom at least 430 million necessitate rehabilitation services. Projections indicate that by 2050, the population affected will rise to 2.5 billion (approximately 1 quarter of the global population), with 700 million of these individuals requiring aural and auditory rehabilitation services. Hearing loss can be categorized according to the frequency range of the sound, into low-frequency, high-frequency and speech-frequency hearing loss. The etiology and pathophysiology of each category differs.^[1–3]^

The etiology of hearing impairment is multifactorial, with well-established risk factors including aging,^[4]^ noise exposure,^[5]^ ototoxic medications,^[6]^ and metabolic disorders,^[7,8]^ such as diabetes and hypertension. In recent years, accumulating evidence suggests that obesity and related anthropometric indices may play a pivotal role in the development of hearing loss.^[9,10]^ Obesity contributes to systemic inflammation, oxidative stress, and vascular dysfunction, all of which are implicated in cochlear microcirculatory damage and auditory dysfunction.^[11,12]^ Traditional obesity indicators, such as body mass index (BMI) and waist circumference (WC), have been extensively studied in relation to hearing loss. However, these measures fail to distinguish between muscle mass and fat distribution, potentially leading to overestimation or underestimation of individual risk, thus limiting their predictive accuracy.^[8,13]^

A novel anthropometric index, the body roundness index (BRI), which incorporates both height and WC, has been proposed as a more precise measure of central obesity and fat distribution compared to BMI. By integrating WC and height, BRI serves as a refined indicator of central adiposity and holds significant potential for clinical applications.^[14,15]^ BRI has been shown to more accurately reflect central obesity and is associated with various metabolic disorders, including cardiovascular diseases,^[16]^ insulin resistance,^[17]^ and systemic inflammation.^[18]^ Given the potential role of adiposity in cochlear dysfunction, investigating the relationship between BRI and hearing loss could provide critical insights into the metabolic impact on auditory impairment. However, evidence exploring this association remains limited.

In order to address this research gap, the objective of this study is to investigate the association between BRI and different types of hearing loss in the United States population using data from the National Health and Nutrition Examination Survey (NHANES).This study employs advanced statistical methods, such as feature selection based on machine learning, in an attempt to elucidate the potential association between BMI and hearing impairment. The findings of this study have the potential to contribute to a more comprehensive understanding of the relationship between obesity and hearing dysfunction, thus providing valuable reference points for the development of future prevention and intervention strategies.

## 2. Materials and methods

### 2.1. Study population

**Figure 1.**
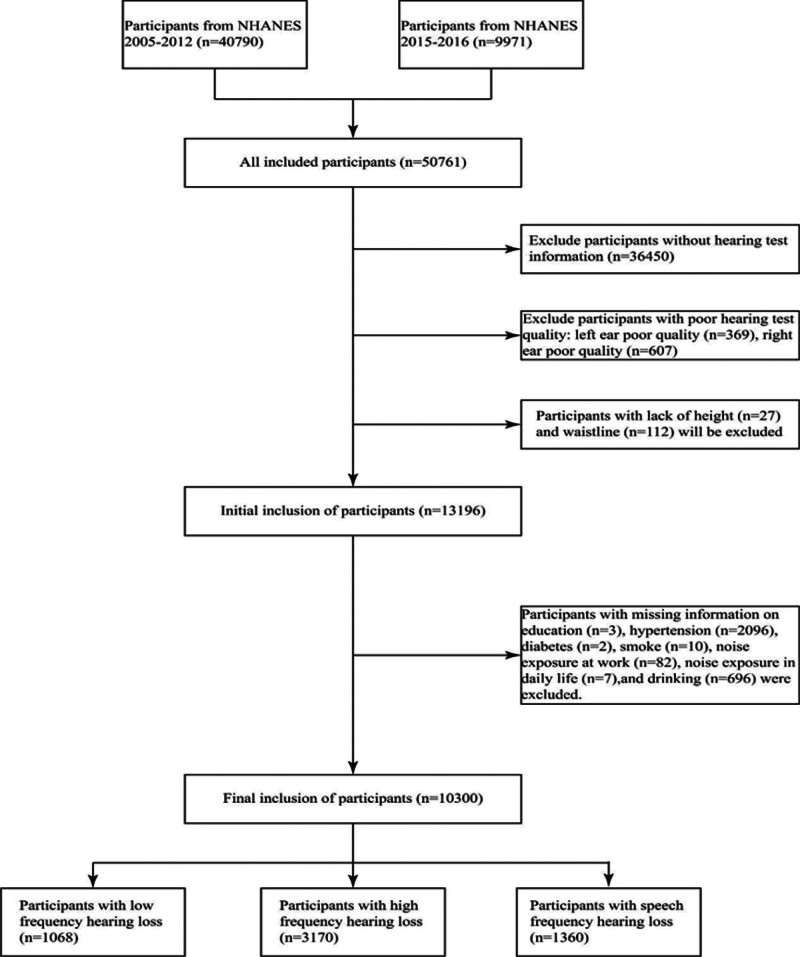
The following flowchart delineates the population included in the present study. NHANES = National Health and Nutrition Examination Survey.

The NHANES, administered by the National Center for Health Statistics (NCHS), is a cross-sectional study designed to assess the health and nutritional status of adults and children in the noninstitutionalized civilian population of the United States. Given the availability of data, the study selected all NHANES years that included hearing-related data (2005–2012 and 2015–2016), with a total of 50,761 participants included in the study. The present study employs the Complete Case Analysis design, adhering to stringent inclusion criteria. The study excluded 40,461 participants who did not meet the following strict exclusion criteria: Firstly, 36,450 participants were excluded on the basis of missing or unknown hearing information. Secondly, 976 participants were excluded based on poor quality of the hearing test in both ears. Thirdly, 27 participants were excluded due to missing or unknown height. Fourthly, 112 participants were excluded based on missing or unknown WC. Finally, participants with missing information on education (n = 3), hypertension (n = 2096), diabetes (n = 2), smoking status (n = 10), alcohol consumption (n = 696), occupational noise exposure (n = 82), and environmental noise exposure (n = 7) were excluded. The final study population included 10,300 participants, comprising 1068 individuals with low-frequency hearing loss, 3170 individuals with high-frequency hearing loss, and 1360 individuals with speech-frequency hearing loss (Fig. 1).

### 2.2. Disease definition

The hearing assessment was conducted in accordance with the standards established by the NCHS. All participants from NHANES were required to undergo a hearing assessment, with the following exceptions: those who could not remove their hearing aids, those who could not tolerate the ear pain caused by the headphones, and those who self-reported ear diseases, excessive cerumen, or abnormalities in otoscopy. Prior to the hearing test, environmental noise levels were measured using a Quest 1800 sound level meter (Quest, Idaho). All examiners were trained to conduct hearing tests at 7 frequencies (500, 1000, 2000, 3000, 4000, 6000, and 8000 Hz) on the participants.^[19]^

The classification of hearing loss in this study encompassed 3 distinct types: low-frequency hearing loss, high-frequency hearing loss, and speech-frequency hearing loss. Low-frequency hearing loss was assessed at 500, 1000, and 2000 Hz; high-frequency hearing loss was assessed at 4000, 6000, and 8000 Hz; and speech-frequency hearing loss was assessed at 500, 1000, 2000, and 4000 Hz. When the overall threshold of the better ear in the various frequency ranges of the 3 types of hearing loss is ≥25 dB, it is considered to have hearing loss in that category.^[20]^

### 2.3. Covariates

A comprehensive review of the extant literature was conducted to identify potential influencing factors,^[21–24]^ which included age (Grouping by the median age of the population), sex, race (Mexican American, Other Hispanic, non-Hispanic White, non-Hispanic Black, Other Race), education (<9th grade, 9–11th grade, high school diploma/GED, some college/AA degree, ≥college graduate), income–poverty ratio, BMI, alcohol consumption, smoking, hypertension, diabetes, and noise exposure at work and in life. Hypertension is defined in 2 ways: first, as a self-reported history of high blood pressure; and second, as the current use of prescribed medication for hypertension. Diabetes is defined as “Doctor told you have diabetes,” “Two Hour Glucose (OGTT; mmol/L) ≥ 11.1,” and “Fasting Glucose (mmol/L) ≥ 7.0.” Prediabetes is defined as an “Ever told you have prediabetes,” “Two Hour Glucose (OGTT; mmol/L) = 7.8–11.1,” and “Fasting Glucose (mmol/L)6.1–6.9.” The classification of smoking status is as follows: individuals with no current smoking history (i.e., those who have never smoked or have quit smoking for >1 year), current smokers (i.e., those who currently smoke or smoked in the past 30 days for >1 day or wake up and smoke or quit smoking and smoke >2 cigarettes/d). The drinking status is categorized as follows: no drinking history (<12 drinks in a lifetime), current drinkers (at least 12 drinks/yr or >6 times in the past 12 months). Participants were considered to be exposed to noise at work or in their personal lives if they responded in the affirmative to the question, “Have you been exposed to loud noise at work or in your personal life?” The BMI of participants was calculated as weight (kg) divided by the square of height (m).

### 2.4. Ethical approval

This study was based on publicly available data from the NHANES, administered by the NCHS. The NHANES protocols were reviewed and approved by the NCHS Research Ethics Review Board, with written informed consent obtained from all participants. As this research involved secondary analysis of de-identified datasets, no additional ethical approval was required.

### 2.5. Statistical analysis

#### 2.5.1. Regression models

Statistical analyses were conducted using R version 4.4.1. Differences between groups were analyzed using design-adjusted chi-square tests and Kruskal–Wallis tests. Logistic regression models were employed to assess the association between BRI and hearing loss. Sequential adjustments were made for covariates across 4 models: model 1: Unadjusted. Model 2: Adjusted for demographic factors (age, sex, race, education, income-to-poverty ratio, BMI). Model 3: Further adjusted for WC, alcohol consumption, smoking, hypertension, and diabetes. Model 4: Additionally adjusted for noise exposure at work and in daily life.

#### 2.5.2. Subgroup analysis

Subgroup analyses were conducted to explore the consistency of the association between BRI and hearing loss across various population groups. Interaction terms were evaluated to determine whether the effect of BRI on hearing loss differed across subgroups. Forest plots were generated to visualize the subgroup-specific ORs and 95% confidence interval (CIs). Statistical significance for interaction terms was assessed using *P*-values.

#### 2.5.3. Restricted cubic spline (RCS) Analysis

Restricted cubic spline (RCS) regression was employed to explore the potential nonlinear relationship between BRI and the predicted probability of hearing loss. Models were adjusted for covariates, including sex, age, race, education, income-to-poverty ratio, BMI, WC, alcohol consumption, smoking, hypertension, diabetes, and noise exposure. The identification of inflection points for each hearing loss type was a key step in the analysis. The calculation of overall and nonlinear *P*-values was undertaken to assess statistical significance. Graphical representations were generated to visualize the relationship between BRI and hearing loss outcomes.

### 2.6. Machine learning approaches

Machine learning models were implemented to enhance the analysis of associations between BRI and hearing loss. Three models were employed: least absolute shrinkage and selection operator (LASSO) Regression: LASSO regression was utilized for variable selection and regularization, with the regularization parameter (λ) being optimized using cross-validation. Variables with non-zero coefficients were retained for model interpretation. Random forest (RF): RF, an ensemble learning method, was applied to identify key predictors and assess variable importance. The performance of the model was evaluated using OOB error rates and the area under the receiver operating characteristic curve (AUC). eXtreme Gradient Boosting (XGBoost): XGBoost, a gradient boosting framework, was utilized to refine predictions through iterative optimization of hyper-parameters to maximize accuracy and interpretability. Shapley additive explanations (SHAP) Analysis: SHAP values were calculated to interpret the contributions of individual predictors in machine learning models. This approach yielded insights into the relative importance and direction of variable effects.

### 2.7. Model validation

All models underwent cross-validation to ensure robustness. Performance metrics, including AUC, were used to compare models. The integration of LASSO, RF, and XGBoost allowed for comprehensive analysis and reliable identification of key predictors of hearing loss.

## 3. Results

### 3.1. Baseline characteristics of participants

A total of 10,300 participants were included in the final analysis. Among them, 10.4% had low-frequency hearing loss, 30.8% had high-frequency hearing loss, and 13.2% had speech-frequency hearing loss. Baseline characteristics showed significant differences in age, sex, race/ethnicity, BMI categories, and exposure to noise at work (*P* < .05 for all). Older participants (≥41 years) and males were more likely to experience hearing loss compared to their counterparts (Tables 1, S1 and S2, Supplemental Digital Content, https://links.lww.com/MD/Q33).

**Table 1:** Baseline characteristics of participants with low-frequency hearing loss.

Characteristic	N*	Overall N = 7,64,03,479†	No N = 6,99,68,888†	Yes N = 64,34,592†	*P*-value‡
Sex	10,297				.713
Female		5075 (51%)	4569 (51%)	506 (51%)	
Male		5222 (49%)	4660 (49%)	562 (49%)	
Age	10,297				<.001
<41		5095 (45%)	5060 (49%)	35 (4.3%)	
≥41		5202 (55%)	4169 (51%)	1033 (96%)	
Race	10,297				<.001
Mexican		1719 (8.5%)	1587 (8.8%)	132 (5.0%)	
Other Hispanic		1029 (5.8%)	961 (6.1%)	68 (2.8%)	
Non-Hispanic White		3872 (67%)	3223 (66%)	649 (80%)	
Non-Hispanic Black		2477 (11%)	2323 (12%)	154 (7.2%)	
Other race		1200 (7.1%)	1135 (7.3%)	65 (5.0%)	
Education	10,297				<.001
<9th		808 (4.5%)	596 (3.8%)	212 (12%)	
9–11th		3144 (20%)	2953 (20%)	191 (16%)	
High School		1815 (18%)	1545 (18%)	270 (26%)	
Some College		2469 (29%)	2231 (29%)	238 (28%)	
College Graduate		2061 (28%)	1904 (29%)	157 (19%)	
Income–poverty ratio	10,297				<.001
<1.3		3212 (21%)	2887 (21%)	325 (21%)	
1.3–3.5		4414 (40%)	3881 (40%)	533 (50%)	
≥3.5		2671 (39%)	2461 (40%)	210 (29%)	
BMI	10,297				.011
<25		3647 (33%)	3353 (34%)	294 (28%)	
25–30		3147 (31%)	2756 (31%)	391 (34%)	
≥30		3503 (35%)	3120 (35%)	383 (38%)	
Waistline	10,297	98.03 ± (16.97)	97.70 ± (17.08)	101.57 ± (15.24)	<.001
Hypertension	10,297				<.001
Without Hypertension		7212 (72%)	6777 (74%)	435 (47%)	
With Hypertension		3085 (28%)	2452 (26%)	633 (53%)	
Diabetes	10,297				<.001
No		6720 (67%)	6174 (69%)	546 (53%)	
Yes		1240 (10%)	962 (8.9%)	278 (23%)	
Borderline		2337 (23%)	2093 (22%)	244 (24%)	
Smoking	10,297				.253
No		7821 (78%)	7004 (78%)	817 (76%)	
Yes		2476 (22%)	2225 (22%)	251 (24%)	
Alcohol consumption	10,297				.917
No		3436 (22%)	3166 (22%)	270 (22%)	
Yes		6861 (78%)	6063 (78%)	798 (78%)	
Noise at work	10,297				<.001
No		7223 (68%)	6582 (69%)	641 (60%)	
Yes		3074 (32%)	2647 (31%)	427 (40%)	
Noise in life	10,297				.811
No		8560 (84%)	7651 (84%)	909 (84%)	
Yes		1737 (16%)	1578 (16%)	159 (16%)	
BRI	10,297	5.22 ± (2.38)	5.16 ± (2.38)	5.91 ± (2.20)	<.001
BRI group	10,297				<.001
1		2574 (23%)	2492 (24%)	82 (7.3%)	
2		2572 (26%)	2303 (26%)	269 (28%)	
3		2576 (26%)	2230 (25%)	346 (31%)	
4		2575 (25%)	2204 (24%)	371 (34%)	

This table presents the baseline characteristics of participants stratified by the presence or absence of low-frequency hearing loss. Variables include demographic information (e.g., sex, age, race), clinical parameters (e.g., BMI, waist circumference, hypertension, diabetes), lifestyle factors (e.g., smoking, alcohol consumption, noise exposure), and the Body Roundness Index (BRI). Statistical significance was assessed using Pearson’s chi-square test for categorical variables and the Kruskal–Wallis test for continuous variables. BRI and age were significantly associated with low-frequency hearing loss (*P* < .001).

BMI = body mass index, BRI = body roundness index.

* N not missing (unweighted).

† n (unweighted; %); mean± (SD).

‡ Pearson’s *X*^2^: Rao & Scott adjustment; design-based Kruskal–Wallis test.

## 3.2. Association of BRI and hearing loss

### 3.2.1. Low-frequency hearing loss

BRI as a continuous variable: Across all models, higher BRI values were significantly associated with increased odds of low-frequency hearing loss. In the fully adjusted model (model 4), the OR for BRI was 1.34 (95% CI: 1.13–1.58, *P* < .001).

BRI quartiles: A dose–response relationship was observed. Compared to the reference group (Q1), the odds of low-frequency hearing loss increased progressively from Q2 (OR: 2.06, 95% CI: 1.34–3.15) to Q4 (OR: 3.58, 95% CI: 1.97–6.48) in model 4. *P* for trend across quartiles was <.001, confirming a statistically significant pattern. The inclusion of additional covariates across models did not substantially weaken the associations, underscoring the robustness of BRI as a predictor (Table 2).

**Table 2:** Association between BRI and low-frequency hearing loss.

Variables	Model 1		Model 2		Model 3		Model 4	
	OR (95% CI)	*P*	OR (95% CI)	*P*	OR (95% CI)	*P*	OR (95% CI)	*P*
BRI	1.12 (1.10–1.15)	<.001	1.39 (1.19–1.64)	.001	1.33 (1.13–1.57)	.001	1.34 (1.13–1.58)	<.001
BRI group								
Q1	1.00 (Reference)		1.00 (Reference)		1.00 (Reference)		1.00 (Reference)	
Q2	3.59 (2.56–5.05)	<.001	2.09 (1.36–3.20)	.001	2.04 (1.34–3.12)	.001	2.06 (1.34–3.15)	.001
Q3	4.09 (2.93–5.71)	<.001	2.34 (1.43–3.83)	<.001	2.20 (1.35–3.58)	.002	2.21 (1.36–3.57)	.001
Q4	4.74 (3.59–6.28)	<.001	4.01 (2.21–7.27)	<.001	3.55 (1.96–6.46)	.001	3.58 (1.97–6.48)	<.001
*P* for trend	<.001		<.001		<.001		<.001	

Model 1: Crude.

Model 2: Adjust: Sex, age, race, education, income–poverty ratio, BMI.

Model 3: Adjust: Sex, age, race, education, income–poverty ratio, BMI, waistline, alcohol consumption, smoking, hypertension, diabetes.

Model 4: Adjust: Sex, age, race, education, income–poverty ratio, BMI, waistline, alcohol consumption, smoking, hypertension, diabetes, noise at work, noise in life.

BMI = body mass index, BRI = body roundness index, CI = confidence interval, OR = odds ratio.

### 3.2.2. High-frequency hearing loss

BRI as a continuous variable: In model 4, the OR was 1.44 (95% CI: 1.32–1.57, *P* < .001), indicating a strong and consistent association between BRI and high-frequency hearing loss.

BRI quartiles: Q4 participants had an OR of 4.85 (95% CI: 3.31–7.11), demonstrating the highest risk among quartiles in model 4. The risk increased progressively across quartiles, with a significant *P* for trend (<.001) in all models. The findings suggest that BRI plays a pivotal role in high-frequency hearing loss, even after adjusting for extensive covariates (Table S3, Supplemental Digital Content, https://links.lww.com/MD/Q33).

### 3.2.3. Speech-frequency hearing loss

BRI as a continuous variable: A significant association was observed, with an OR of 1.46 (95% CI: 1.27–1.68, *P* < .001) in the fully adjusted model.

BRI quartiles: Participants in Q4 had an OR of 5.00 (95% CI: 2.96–8.43) compared to the reference (Q1), indicating a pronounced risk. A dose–response relationship was evident, supported by *P* for trend values <.001 in all models. These results underline the critical influence of BRI on speech-frequency hearing loss, further validated by the consistent findings across models (Table S4, Supplemental Digital Content, https://links.lww.com/MD/Q33).

For all hearing loss types. Consistent Positive Association: BRI, whether treated as a continuous variable or stratified into quadrilles, showed a strong positive relationship with the odds of hearing loss.

Dose–response relationship: Quartile analysis revealed increasing ORs across BRI categories, with the highest quartile consistently exhibiting the greatest risk.

Robustness: The associations remained statistically significant across models, even with extensive adjustments for demographic, clinical, and environmental excoriates.

## 3.3. Subgroup analysis

### 3.3.1. Low-frequency hearing loss

Loss-in the subgroup analysis of low-frequency hearing loss, the association between BRI and hearing impairment remained significant across most subgroups, with some variations in effect size:

The association was found to be more pronounced in participants aged 41 years and above (odds ratio [OR] = 1.34, 95% CI: 1.13–1.58, *P* = .001) compared to those younger than 41 years (OR = 1.26, 95% CI: 0.66–2.38, *P* = .477). This finding indicates that the impact of BRI on low-frequency hearing loss is more evident in older subjects.

Sex: The association was significant in males (OR = 2.19, 95% CI: 1.70–2.82, *P* < .001) but not in females (OR = 1.08, 95% CI: 0.90–1.31, *P* = .407). These findings suggest the possibility of sex-related differences in vulnerability to hearing loss associated with body roundness.

Race: Among the various racial groups, the strongest associations were observed in Mexican Americans (OR = 1.57, 95% CI: 1.22–2.02, *P* < .001) and non-Hispanic Whites (OR = 1.56, 95% CI: 1.25–1.95, *P* < .001), while the association was weaker in non-Hispanic Blacks (OR = 1.27, 95% CI: 1.05–1.53, *P* = .014).

Education level: Individuals with a college degree exhibited a weaker association compared to those with lower education levels (OR = 1.37, 95% CI: 0.97–1.94, *P* = .072).

In the context of diabetes, the association was found to be statistically significant (OR = 1.47, 95% CI: 1.23–1.77, *P* < .001). The association remained statistically significant among participants with diabetes (OR = 1.47, 95% CI: 1.23–1.77, *P* < .001), but was not significant among those with borderline diabetes (*P* = .704).

Hypertension: Individuals with hypertension exhibited a higher risk (OR = 1.53, 95% CI: 1.17–1.99, *P* = .002) compared to those without (OR = 1.33, 95% CI: 1.08–1.64, *P* = .009).

In the context of noise exposure, the impact of BRI manifested with greater potency among individuals exposed to occupational noise (OR = 1.71, 95% CI: 1.13–2.59, *P* = .007) in comparison to those lacking exposure.

**Figure 2.**
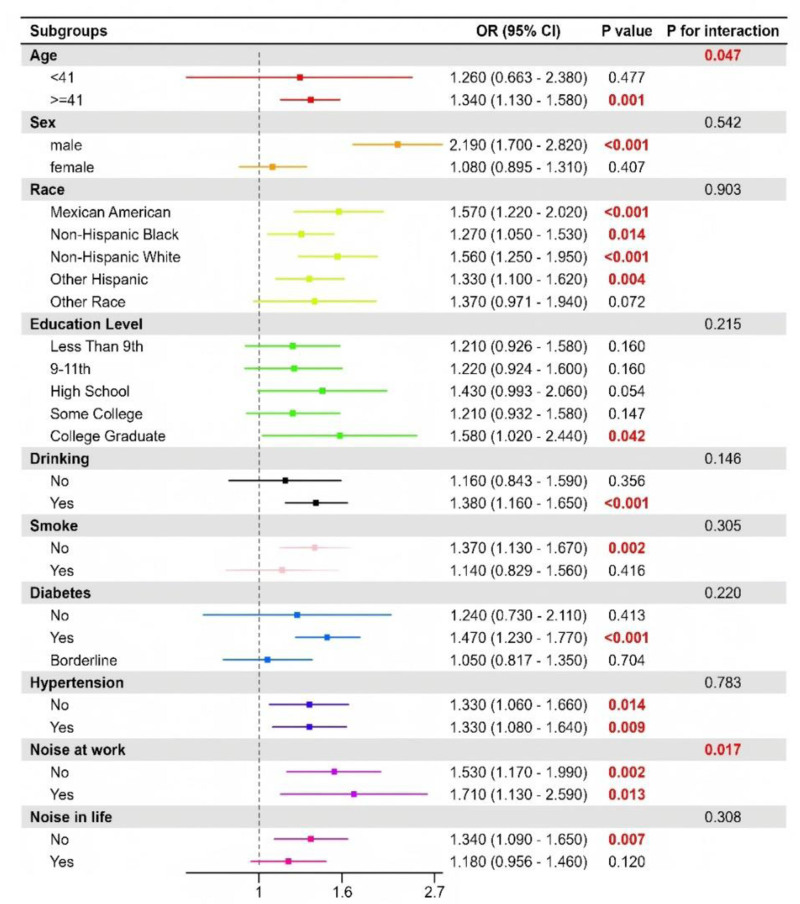
Subgroup analysis of association between BRI and low-frequency hearing loss. BRI = body roundness index, CI = confidence interval, OR = odds ratio.

The interaction terms for sex, hypertension and occupational noise were statistically significant, suggesting potential modifying effects (Fig. 2).

### 3.3.2. High-frequency hearing loss

Loss-for high-frequency hearing loss, BRI exhibited a strong and consistent association across most subgroups:

Age: The association was stronger in older adults (≥41 years; OR = 1.44, 95% CI: 1.31–1.58, *P* < .001) than in younger adults (<41 years; OR = 1.40, 95% CI: 1.14–1.72, *P* = .002).

Sex: A stronger association was observed in males (OR = 1.97, 95% CI: 1.55–2.50, *P* < .001) than in females (OR = 1.16, 95% CI: 1.04–1.29, *P* = .007).

Race: The highest risk was observed among non-Hispanic Whites (OR = 1.51, 95% CI: 1.24–1.85, *P* < .001), while Mexican Americans (OR = 1.42, 95% CI: 1.07–1.88, *P* = .016) also demonstrated a significant association.

Diabetes: The association was particularly strong among diabetic individuals (OR = 2.00, 95% CI: 1.51–2.65, *P* < .001).

Hypertension: The presence of hypertension significantly amplified the risk (OR = 1.66, 95% CI: 1.34–2.06, *P* < .001).

Furthermore, individuals exposed to workplace noise had a higher risk (OR = 1.55, 95% CI: 1.25–1.92, *P* < .001).

The interaction terms for diabetes and hypertension were found to be significant, suggesting that metabolic conditions may modulate the relationship between BRI and high-frequency hearing loss (Fig. S1, Supplemental Digital Content, https://links.lww.com/MD/Q32).

### 3.3.3. Speech-frequency hearing loss

Loss-the subgroup analysis for speech-frequency hearing loss revealed the following patterns:

Age: The association was significant in older adults (≥41 years; OR = 1.46, 95% CI: 1.26–1.69, *P* < .001) but not in younger adults (<41 years; OR = 1.47, 95% CI: 0.91–2.37, *P* = .118).

Sex: The association was stronger in males (OR = 2.01, 95% CI: 1.63–2.48, *P* < .001) compared to females (OR = 1.15, 95% CI: 0.97–1.38, *P* = .112).

In terms of race, Mexican Americans (OR = 1.49, 95% CI: 1.15–1.95, *P* = .004) and non-Hispanic Whites (OR = 1.62, 95% CI: 1.36–1.93, *P* < .001) demonstrated significant associations.

Education level: The risk was found to be highest among individuals with lower levels of education.

Diabetes: A significant association was observed among diabetic participants (OR = 1.65, 95% CI: 1.37–1.99, *P* < .001).

Hypertension: Individuals with hypertension demonstrated a stronger association (OR = 1.66, 95% CI: 1.26–2.17, *P* < .001).

The impact of noise exposure on the relationship was found to be significantly modified by the workplace environment (OR = 1.65, 95% CI: 1.19–2.29, *P* = .003).

These findings underscore the necessity of incorporating age, sex, race, metabolic conditions, and environmental factors into the evaluation of the impact of BRI on speech-frequency hearing loss (Fig. S2, Supplemental Digital Content, https://links.lww.com/MD/Q32).

Conclusion across all 3 types of hearing loss, BRI remained a significant predictor, with stronger effects observed in older adults, males, individuals with diabetes or hypertension, and those exposed to occupational noise. The findings underscore the potential for biological and environmental interactions that influence hearing impairment.

### 3.3.4. Restricted cubic spline analysis

**Figure 3.**
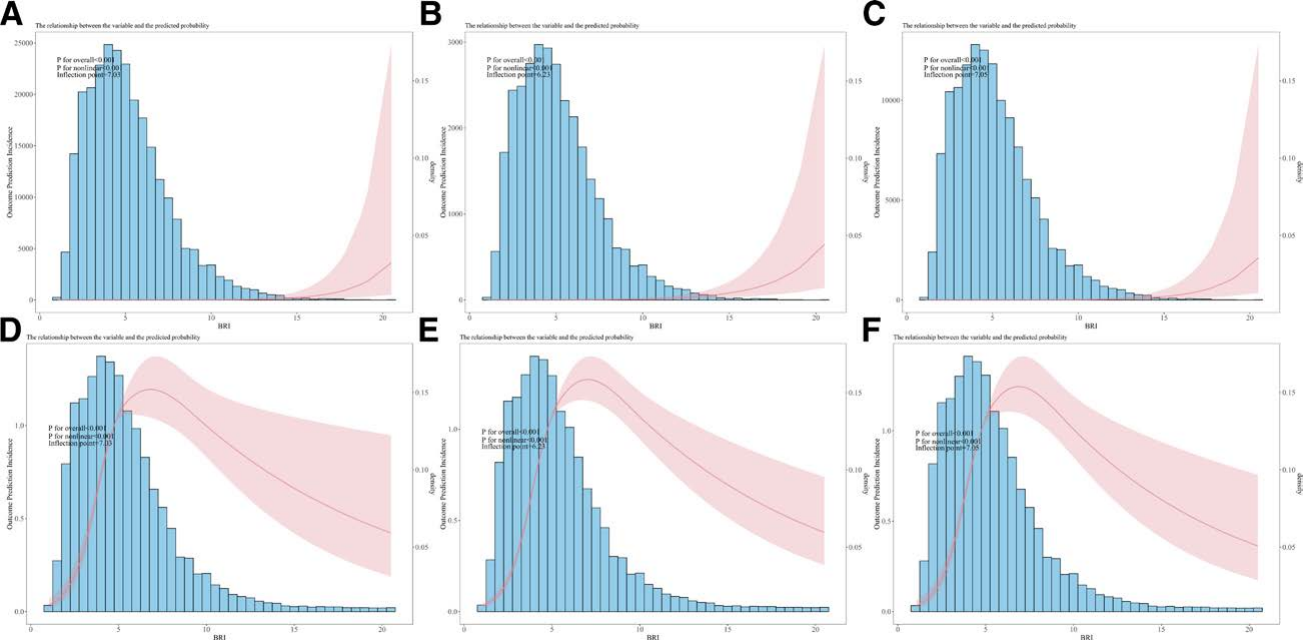
Restricted cubic spline model of the association between BRI and Hearing Loss. Panels A, B, and C: Restricted cubic spline (RCS) models with full adjustment for covariates, showing associations between BRI and the predicted probability of low-frequency (A), high-frequency (B), and speech-frequency (C) hearing loss. Panels D, E, and F: Corresponding unadjusted models for low-frequency (D), high-frequency (E), and speech-frequency (F) hearing loss. The *x*-axis represents BRI, while the *y*-axis denotes the predicted probability or incidence density of hearing loss. The dashed vertical line indicates the inflection point where the relationship shifts, noted as 7.03 for low-frequency, 6.23 for high-frequency, and 7.05 for speech-frequency hearing loss. BRI = body roundness index, RCS = restricted cubic spline.

RCS analysis revealed nonlinear relationships between BRI and the predicted probability of hearing loss for all frequency domains. For all hearing loss types: Below the inflection points, the relationship between BRI and hearing loss exhibited steeper increases in risk. Above the inflection points, the risk plateaued but remained significant. Overall and nonlinear *P*-values were <.001 for all models, confirming the robustness of the nonlinear associations. Graphical representations were employed to elucidate the dose–response relationships, underscoring the pivotal role of BRI in predicting hearing loss outcomes (Fig. 3).

## 3.4. Threshold effect analysis for BRI and hearing loss

### 3.4.1. Low-frequency hearing loss

One-line linear regression model: The OR for the relationship between BRI and low-frequency hearing loss was 1.67 (95% CI: 1.42–1.97, *P* < .001), indicating a significant positive association.

Two-piece-wise linear regression model:

Inflection point: 7.03

Below the inflection point (<7.03), the OR was 2.08 (95% CI: 1.80–2.42, *P* < .001), suggesting a stronger association. Above the inflection point (>7.03), the OR was 1.56 (95% CI: 1.38–1.77, *P* < .001), indicating a weaker but still significant association. The log-likelihood ratio test (*P* = .001) confirmed that the 2-piece-wise model fit the data better than the 1-line model, highlighting a threshold effect (Table 3).

**Table 3:** Threshold effect analysis of BRI and low-frequency hearing loss.

	OR (95% CI)	*P*-value
1-line linear regression model	1.67 (1.42–1.97)	<.001
2-piece-wise linear regression model		
Inflection point	7.03	
<7.03	2.08 (1.80–2.42)	<.001
>7.03	1.56 (1.38–1.77)	<.001
Log-likelihood ratio test		.001

Sex, age, race, education, income–poverty ratio, BMI, waistline, alcohol consumption, smoking, hypertension, diabetes, noise at work, noise in life.

BMI = body mass index, BRI = body roundness index, CI = confidence interval, OR = odds ratio.

### 3.4.2. High-frequency hearing loss

One-line linear regression model: The OR for BRI and high-frequency hearing loss was 1.46 (95% CI: 1.27–1.68, *P* < .001), showing a significant positive relationship.

Two-piece-wise linear regression model:

Inflection point: 6.23

Below the inflection point (<6.23), the OR was 2.10 (95% CI: 1.76–2.27, *P* < .001), indicating a stronger association. Above the inflection point (>6.23), the OR was 1.46 (95% CI: 1.33–1.60, *P* < .001), showing a reduced but significant relationship. The log-likelihood ratio test (*P* = .001) supported the 2-piece-wise model, suggesting a nonlinear association (Table S5, Supplemental Digital Content, https://links.lww.com/MD/Q33).

### 3.4.3. Speech-frequency hearing loss

One-line linear regression model: The OR for BRI and speech-frequency hearing loss was 1.66 (95% CI: 1.42–1.93, *P* < .001), reflecting a significant positive relationship.

Two-piece-wise linear regression model:

Inflection point: 7.05.

Below the inflection point (<7.05), the OR was 2.02 (95% CI: 1.76–2.32, *P* < .001), suggesting a strong association. Above the inflection point (>7.05), the OR was 1.52 (95% CI: 1.36–1.71, *P* < .001), indicating a weaker yet significant relationship. The log-likelihood ratio test (*P* = .001) confirmed the superiority of the 2-piece-wise model in explaining the relationship (Table S6, Supplemental Digital Content, https://links.lww.com/MD/Q33).

Across all hearing loss types, the relationship between BRI and hearing loss demonstrated a threshold effect: The associations were stronger below the respective inflection points but remained significant above them. The 2-piece-wise linear regression models provided a better fit compared to the 1-line models. Covariates such as sex, age, race, education, income–poverty ratio, BMI, WC, alcohol consumption, smoking, hypertension, diabetes, and noise exposure were adjusted in all analyses.

## 3.5. Feature selection and model optimization

### 3.5.1. LASSO regression

**Figure 4.**
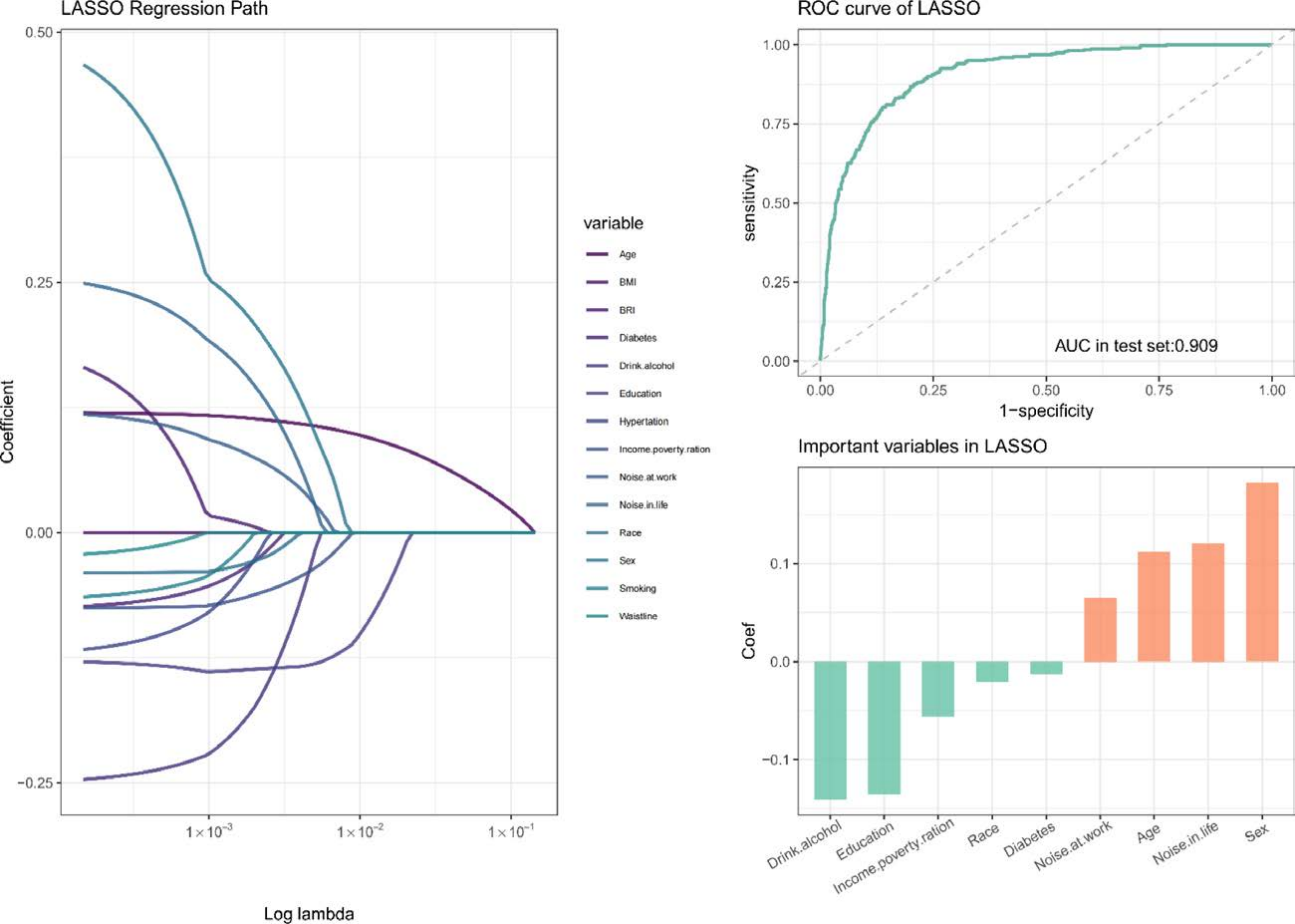
LASSO regression model for low-frequency hearing loss. AUC = area under the curve, LASSO = least absolute shrinkage and selection operator, ROC = receiver operating characteristic.

LASSO regression was employed to identify key predictors for low-frequency, high-frequency, and speech-frequency hearing loss, effectively controlling for multidisciplinary. The penalty parameter, designated as λ, was optimized through the implementation of cross-validation, thereby ensuring a balance between model complexity and predictive performance (Figs. S3–S5, Supplemental Digital Content, https://links.lww.com/MD/Q32). The model’s performance was evaluated across all 3 types of hearing loss:

Low-frequency hearing loss: The identification of significant predictors, such as BRI, age, BMI, and WC, underscores the comprehensive nature of the assessment. The AUC for the LASSO model was 0.909, indicating high predictive accuracy (Fig. 4).

High-frequency hearing loss: BRI, age, BMI, and noise exposure at work emerged as critical variables, achieving an AUC of 0.935 (Fig. S6, Supplemental Digital Content, https://links.lww.com/MD/Q32).

Speech-frequency hearing loss: The key predictors identified were BRI, age, BMI, and noise exposure at work, with an AUC of 0.917. The coefficient path plots for each model demonstrated the shrinkage effect on less important variables, ensuring parsimonious and interpretable models (Fig. S7, Supplemental Digital Content, https://links.lww.com/MD/Q32).

### 3.5.2. Random forest. 

**Figure 5.**
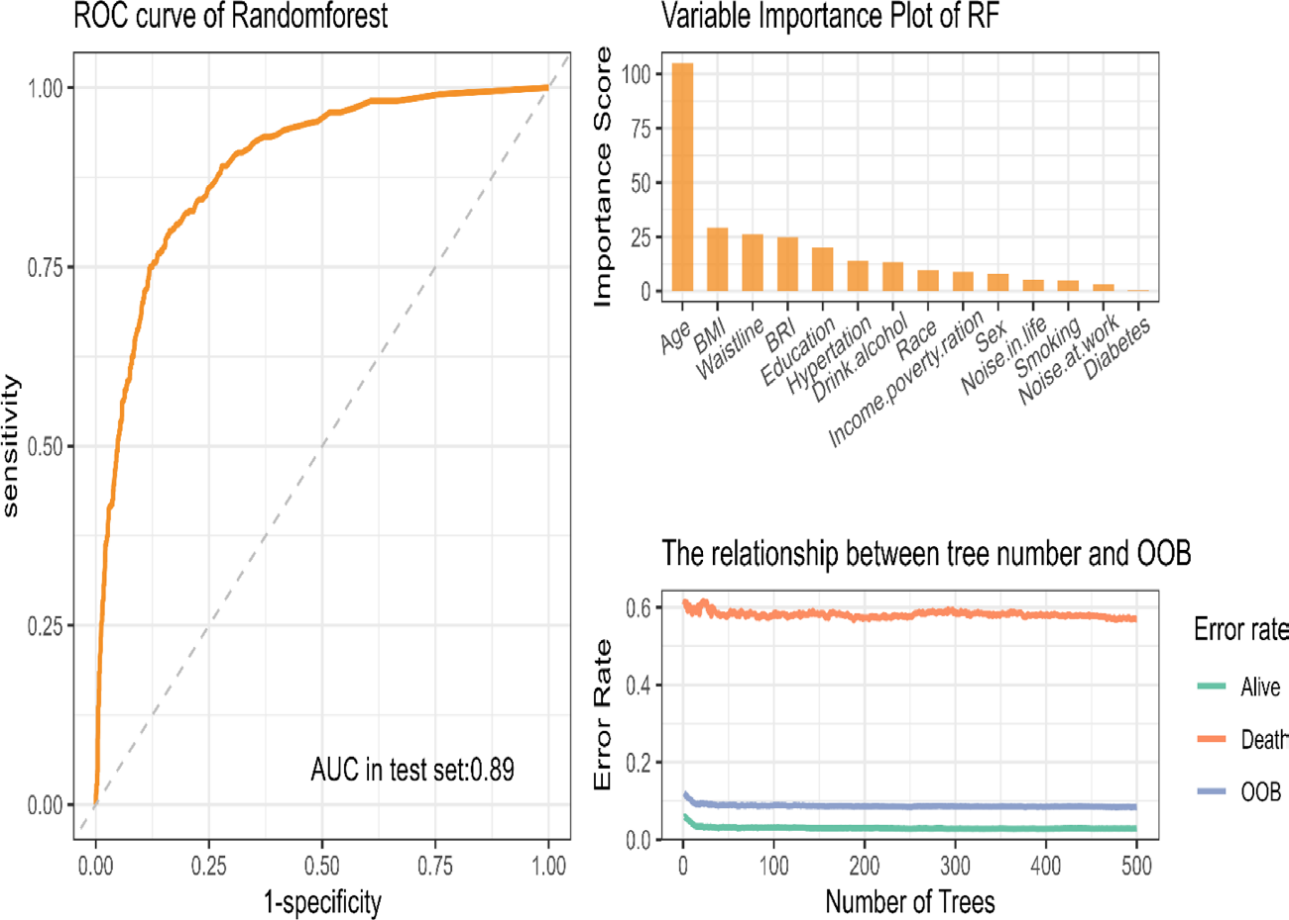
Random forest model for low-frequency hearing loss. AUC = area under the curve, OOB = out-of-bag, RF = random forest, ROC = receiver operating characteristic.

RF models were capable of capturing nonlinear relationships and interactions among predictors. The following key findings emerged from this analysis:

Low-frequency hearing loss: The top predictors were identified as age, BMI, WC, and BRI. The model attained an AUC of 0.89, with its performance substantiated by OOB error rates (Fig. 5).

In the context of high-frequency hearing loss, the following predictors emerged as particularly salient: Variable importance rankings identified BRI, BMI, and age as critical factors. The AUC was 0.93, underscoring the model’s strong predictive capacity (Fig. S8, Supplemental Digital Content, https://links.lww.com/MD/Q32).

Speech-frequency hearing loss: BRI, BMI, and noise exposure at work were among the top predictors, achieving an AUC of 0.907. These findings underscore the robustness and adaptability of RF for analyzing a range of hearing loss outcomes (Fig. S9, Supplemental Digital Content, https://links.lww.com/MD/Q32).

### 3.5.3. eXtreme Gradient Boosting

**Figure 6.**
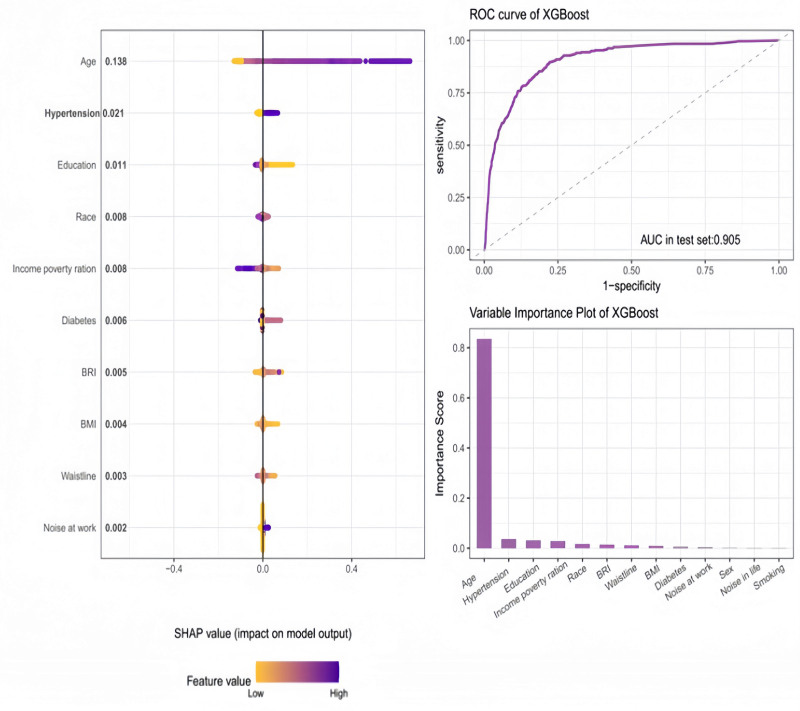
Predictive model performance and feature importance for low-frequency hearing loss. AUC = area under the curve, BMI = body mass index, BRI = body roundness index, ROC = receiver operating characteristic, SHAP = Shapley additive explanations, XGBoost = eXtreme Gradient Boosting.

XGBoost models yielded refined predictions and enhanced interpretability through SHAP analysis. The study’s key findings included the following:

Low-frequency hearing loss: The top predictors were determined to be noise exposure at work, WC, and BMI, with an AUC of 0.905. The significance of BMI, WC, and noise exposure at work is further underscored by the SHAP values (Fig. 6).

In the context of high-frequency hearing loss, the following key findings emerged: SHAP analysis identified diabetes, BMI, and BRI as significant contributors. While diabetes and BMI demonstrated slightly higher rankings, BRI exhibited a consistent influence on model predictions, underscoring its significance in comprehending the risk of high-frequency hearing loss. The model attained an AUC of 0.933, thereby demonstrating its efficacy in predicting high-frequency hearing loss (Fig. S10, Supplemental Digital Content, https://links.lww.com/MD/Q32).

Speech-frequency hearing loss: The analysis identified noise exposure at work, BMI, and the income-to-poverty ratio as critical predictors. However, BRI maintained its position as a consistently influential variable, thereby reinforcing its established role in the prediction of speech-frequency hearing loss. The AUC was 0.911. Furthermore, SHAP plots offered comprehensive insights into the relationships between predictors and hearing loss outcomes (Fig. S11, Supplemental Digital Content, https://links.lww.com/MD/Q32).

As a predictor in this study, the BRI had a significant predictive effect in all 3 types of hearing loss: low-frequency, high-frequency and speech-frequency. Its impact was particularly significant in the following areas: These findings highlight the key role of the BRI in understanding and predicting hearing loss, and demonstrate its potential value in clinical assessments and public health strategies aimed at reducing the risk to auditory health. LASSO regression further emphasized the importance of the BRI, retaining it as a key feature in all models. RF identified the BRI as one of the most important predictors, reflecting its significant contribution to model performance. SHAP analysis in XGBoost showed that the BRI’s impact on prediction was consistent, even when other factors such as BMI or noise exposure were prominent.

## 4. Discussion

In this nationally representative study, a significant association was identified between the BRI and various types of hearing loss, including low-frequency, high-frequency, and speech-frequency hearing impairment. The findings demonstrated a dose–response relationship, where higher BRI values were consistently associated with an increased risk of hearing impairment, even after adjusting for potential confounding factors. Furthermore, the results indicated a nonlinear relationship between BRI and hearing loss, as evidenced by RCS analysis, suggesting a possible threshold effect of BRI on auditory dysfunction. These findings provide new insights into the potential role of obesity and body composition in auditory health, expanding prior research that primarily focused on traditional obesity indices such as BMI and WC.

Several biological mechanisms may underlie the observed association between BRI and hearing loss. First, excessive adiposity, particularly central obesity, is closely linked to vascular dysfunction, which may impair cochlear blood flow and oxygenation, ultimately leading to auditory dysfunction.^[25]^ The cochlea relies on an intricate microvascular network for adequate perfusion, and obesity-related metabolic disorders such as endothelial dysfunction and impaired glucose metabolism may result in cochlear ischemia and subsequent hearing loss.^[26,27]^ Second, chronic low-grade inflammation, a hallmark of obesity, has been linked to auditory dysfunction.^[28]^ Adipose tissue is known to secrete pro-inflammatory cytokines such as tumor necrosis factor-α and interleukin-6, which can disrupt cochlear homeostasis and accelerate the degeneration of auditory sensory cells.^[29,30]^ Elevated inflammatory markers have been associated with both obesity and hearing impairment, supporting the hypothesis that inflammation serves as a critical link between metabolic dysregulation and auditory dysfunction.^[31]^ Third, oxidative stress is a major factor in the onset and progression of hearing loss. Reactive oxygen species can damage cochlear tissue, while antioxidant therapies have shown protective effects in both animal and clinical studies.^[32,33]^ Research has indicated that oxidative stress-induced apoptosis of auditory sensory cells contributes to progressive hearing loss, particularly among individuals with high-fat dietary habits.^[34,35]^ Lastly, insulin resistance and metabolic dysregulation may also contribute to hearing impairment. The insulin-like growth factor-1 signaling pathway plays a crucial role in the temporal differentiation of cochlear hair cells.^[36]^ Studies in insulin receptor substrate 2-deficient mice have demonstrated that sensorineural hearing loss can occur before the onset of diabetes, with reduced cochlear nerve innervation and abnormal stria vascularis, further underscoring the importance of insulin signaling in auditory function.^[37]^

The findings of this study have significant clinical and public health implications. Given the rising global prevalence of obesity and its associated metabolic complications,^[38]^ identifying BRI as a predictor of hearing loss highlights the necessity of an integrated approach to managing metabolic and auditory health. Routine hearing screening in individuals with high BRI may facilitate early detection and intervention, potentially reducing the burden of hearing-related disability. Furthermore, this study underscores the significance of obesity prevention and weight management as a pivotal strategy to mitigate the risk of hearing loss. Lifestyle interventions, including dietary modifications, physical activity, and weight management, have been shown to improve metabolic health,^[39]^ and may also confer protective effects against auditory dysfunction. Public health initiatives should consider incorporating hearing health into obesity prevention programs to address shared risk factors contributing to both metabolic and auditory dysfunction. Future research should further explore the longitudinal impact of BRI on hearing function and investigate potential interventions to reduce obesity-related auditory impairment.

It is imperative that future research endeavors concentrate on longitudinal studies to ascertain the causal relationship between BRI and hearing loss. Prospective cohort studies that meticulously monitor changes in BRI and auditory function over time will be indispensable in determining whether heightened BRI directly contributes to hearing impairment or if the observed associations are confounded by other metabolic factors. Moreover, mechanistic studies that investigate the specific pathways through which obesity affects cochlear function are necessary. The employment of advanced imaging techniques, such as MRI-based cochlear blood flow assessments, holds promise in providing more profound insights into the vascular contributions to hearing impairment. Such investigations would facilitate the elucidation of whether obesity-related microvascular dysfunction is a primary driver of auditory decline. Furthermore, interventional trials assessing the effects of weight reduction and metabolic control on auditory function could provide valuable evidence for potential prevention strategies. Furthermore, studies evaluating the efficacy of lifestyle modifications, such as dietary and physical activity interventions, in mitigating obesity-related hearing loss, would be of significant benefit. The findings from such studies could inform targeted clinical and public health approaches aimed at preserving auditory health in individuals at risk of metabolic disorders. The development of a comprehensive understanding of the metabolic influences on hearing impairment will be enabled by future research integrating multidisciplinary approaches, combining epidemiology, metabolic science, audiology and advanced imaging.

## 5. Strengths and limitations

The present study has several strengths. Firstly, the use of NHANES data provides a large, nationally representative sample, thus enhancing the generalizability of the findings. Secondly, advanced statistical and machine learning techniques were employed, including RCS analysis, LASSO regression, and SHAP-based interpretability models, which allowed for a more comprehensive assessment of the relationship between BRI and hearing loss. Thirdly, a wide range of potential confounders were adjusted for in the study, thereby minimizing bias and enhancing the robustness of the results. However, some limitations should be acknowledged. Firstly, the cross-sectional design of NHANES precludes the establishment of causal inference, and longitudinal studies are needed to establish the temporal relationship between BRI and hearing impairment. Secondly, although BRI is a more refined anthropometric index than BMI, it does not provide direct measures of visceral adiposity, which may have a more pronounced impact on metabolic and auditory health. Thirdly, self-reported lifestyle factors, including noise exposure and smoking, are susceptible to recall bias, which has the potential to compromise the study’s findings. Future research should focus on longitudinal studies to confirm the causality of the relationship between BRI and hearing loss.

## 6. Conclusions

The findings indicate that obesity management should be incorporated into auditory health strategies. Routine screening for hearing loss in individuals with high BRI could facilitate early intervention. Future research should explore the mechanistic pathways linking central obesity to cochlear dysfunction and evaluate whether weight management interventions can mitigate hearing loss risk.

## Acknowledgments

The authors would like to thank all participants in the NHANES database.

## Author contributions

**Conceptualization:** Guoxiong Li, Shangjin Yang.

**Data curation:** Guoxiong Li, Shangjin Yang, Yuzhou Cai.

**Formal analysis:** Guoxiong Li, Shangjin Yang.

**Methodology:** Guoxiong Li, Yuzhou Cai.

**Software:** Guoxiong Li, Shangjin Yang, Yuzhou Cai.

**Supervision:** Shuang Liu, Chengmin Shi, Yujian Zeng.

**Visualization:** Guoxiong Li, Shangjin Yang.

**Writing – original draft:** Guoxiong Li, Shangjin Yang, Haixia Wu.

**Writing – review & editing:** Guoxiong Li, Yuzhou Cai, Haixia Wu.

## Supplementary Material
