# Supplementary material for: Association between body roundness index and hearing loss in the United States population: A cross-sectional study
Source: Medicine (Baltimore). 2025 Oct 3;104(40):e44401. doi: 10.1097/MD.0000000000044401 (PMC12499831; doi:10.1097/MD.0000000000044401)
Supplement: Supplementary file 1 [file medi-104-e44401-s001.docx]

| **Characteristic** | **N**^1^ | **Overall**  N = 76,382,274^2^ | **no**   N = 67,783,682^2^ | **yes**   N = 8,598,591^2^ | **p-value**^3^ |
| --- | --- | --- | --- | --- | --- |
| Sex | 10,292 |  |  |  | <0.001 |
| Female |  | 5,072 (51%) | 4,502 (51%) | 570 (44%) |  |
| Male |  | 5,220 (49%) | 4,430 (49%) | 790 (56%) |  |
| Age | 10,292 |  |  |  | <0.001 |
| <41 |  | 5,094 (45%) | 5,058 (51%) | 36 (3.4%) |  |
| >=41 |  | 5,198 (55%) | 3,874 (49%) | 1,324 (97%) |  |
| Race | 10,292 |  |  |  | <0.001 |
| Mexican |  | 1,718 (8.5%) | 1,540 (8.9%) | 178 (5.3%) |  |
| Other Hispanic |  | 1,028 (5.8%) | 926 (6.2%) | 102 (3.2%) |  |
| Non-Hispanic White |  | 3,870 (67%) | 3,054 (65%) | 816 (81%) |  |
| Non-Hispanic Black |  | 2,476 (11%) | 2,287 (12%) | 189 (6.4%) |  |
| Other Race |  | 1,200 (7.1%) | 1,125 (7.4%) | 75 (4.5%) |  |
| Education | 10,292 |  |  |  | <0.001 |
| Less Than 9th |  | 807 (4.5%) | 548 (3.7%) | 259 (11%) |  |
| 9-11th |  | 3,144 (20%) | 2,904 (20%) | 240 (15%) |  |
| High School |  | 1,812 (18%) | 1,457 (17%) | 355 (26%) |  |
| Some College |  | 2,469 (29%) | 2,167 (29%) | 302 (28%) |  |
| College Graduate |  | 2,060 (28%) | 1,856 (30%) | 204 (19%) |  |
| Income-poverty ration | 10,292 |  |  |  | <0.001 |
| <1.3 |  | 3,211 (21%) | 2,804 (21%) | 407 (19%) |  |
| 1.3-3.5 |  | 4,411 (40%) | 3,742 (39%) | 669 (49%) |  |
| >=3.5 |  | 2,670 (39%) | 2,386 (40%) | 284 (32%) |  |
| BMI | 10,292 |  |  |  | <0.001 |
| <25 |  | 3,647 (33%) | 3,287 (34%) | 360 (27%) |  |
| 25-30 |  | 3,144 (31%) | 2,639 (31%) | 505 (35%) |  |
| ≥30 |  | 3,501 (35%) | 3,006 (35%) | 495 (38%) |  |
| Waistline | 10,292 | 98.03± (16.97) | 97.48± (17.11) | 102.35± (15.12) | <0.001 |
| Hypertension | 10,292 |  |  |  | <0.001 |
| without Hypertension |  | 7,210 (72%) | 6,648 (74%) | 562 (50%) |  |
| with Hypertension |  | 3,082 (28%) | 2,284 (26%) | 798 (50%) |  |
| Diabetes | 10,292 |  |  |  | <0.001 |
| No |  | 6,716 (67%) | 6,018 (69%) | 698 (55%) |  |
| Yes |  | 1,240 (10%) | 877 (8.5%) | 363 (23%) |  |
| Borderline |  | 2,336 (23%) | 2,037 (23%) | 299 (23%) |  |
| Smoking | 10,292 |  |  |  | 0.193 |
| No |  | 7,817 (78%) | 6,786 (78%) | 1,031 (75%) |  |
| Yes |  | 2,475 (22%) | 2,146 (22%) | 329 (25%) |  |
| Alcohol consumption | 10,292 |  |  |  | 0.015 |
| No |  | 3,435 (22%) | 3,132 (22%) | 303 (18%) |  |
| Yes |  | 6,857 (78%) | 5,800 (78%) | 1,057 (82%) |  |
| Noise at work | 10,292 |  |  |  | <0.001 |
| No |  | 7,221 (68%) | 6,433 (69%) | 788 (58%) |  |
| Yes |  | 3,071 (32%) | 2,499 (31%) | 572 (42%) |  |
| Noise in life | 10,292 |  |  |  | 0.892 |
| No |  | 8,556 (84%) | 7,410 (84%) | 1,146 (83%) |  |
| Yes |  | 1,736 (16%) | 1,522 (16%) | 214 (17%) |  |
| BRI | 10,292 | 5.22± (2.38) | 5.14± (2.39) | 5.88± (2.14) | <0.001 |
| BRI group | 10,292 |  |  |  | <0.001 |
| 1 |  | 2,573 (23%) | 2,471 (25%) | 102 (7.4%) |  |
| 2 |  | 2,572 (26%) | 2,245 (26%) | 327 (25%) |  |
| 3 |  | 2,574 (26%) | 2,116 (25%) | 458 (34%) |  |
| 4 |  | 2,573 (25%) | 2,100 (24%) | 473 (34%) |  |
| ^1^N not Missing (unweighted) | | | | | |
| ^2^n (unweighted) (%); Mean± (SD) | | | | | |
| ^3^Pearson's X^2: Rao & Scott adjustment; Design-based KruskalWallis test | | | | | |

**Supplementary Table 1:** Baseline Characteristics of Participants with High-Frequency Hearing Loss

This table summarizes the baseline characteristics of participants categorized by the presence of high-frequency hearing loss. Key variables include demographic data, clinical measures, lifestyle factors, and BRI. Statistical significance for categorical variables was evaluated using Pearson’s chi-square test, while continuous variables were assessed with the Kruskal-Wallis test. Significant differences were noted in age, sex, hypertension, and noise exposure at work, with higher BRI values observed in individuals with high-frequency hearing loss (p < 0.001).

| **Characteristic** | **N**^1^ | **Overall**  N = 76,123,513^2^ | **no**   N = 52,400,369^2^ | **yes**   N = 23,723,144^2^ | **p-value**^3^ |
| --- | --- | --- | --- | --- | --- |
| Sex | 10,242 |  |  |  | <0.001 |
| Female |  | 5,060 (51%) | 3,677 (54%) | 1,383 (44%) |  |
| Male |  | 5,182 (49%) | 3,395 (46%) | 1,787 (56%) |  |
| Age | 10,242 |  |  |  | <0.001 |
| <41 |  | 5,093 (46%) | 4,906 (63%) | 187 (6.8%) |  |
| >=41 |  | 5,149 (54%) | 2,166 (37%) | 2,983 (93%) |  |
| Race | 10,242 |  |  |  | <0.001 |
| Mexican |  | 1,711 (8.5%) | 1,270 (9.6%) | 441 (6.1%) |  |
| Other Hispanic |  | 1,025 (5.8%) | 739 (6.7%) | 286 (3.8%) |  |
| Non-Hispanic White |  | 3,837 (67%) | 2,241 (62%) | 1,596 (77%) |  |
| Non-Hispanic Black |  | 2,474 (12%) | 1,902 (13%) | 572 (7.4%) |  |
| Other Race |  | 1,195 (7.1%) | 920 (7.9%) | 275 (5.2%) |  |
| Education | 10,242 |  |  |  | <0.001 |
| Less Than 9th |  | 796 (4.5%) | 338 (3.2%) | 458 (7.4%) |  |
| 9-11th |  | 3,138 (20%) | 2,597 (23%) | 541 (13%) |  |
| High School |  | 1,798 (18%) | 1,002 (15%) | 796 (25%) |  |
| Some College |  | 2,460 (29%) | 1,666 (28%) | 794 (29%) |  |
| College Graduate |  | 2,050 (29%) | 1,469 (30%) | 581 (25%) |  |
| Income-poverty ration | 10,242 |  |  |  | 0.031 |
| <1.3 |  | 3,199 (21%) | 2,273 (22%) | 926 (19%) |  |
| 1.3-3.5 |  | 4,381 (40%) | 2,904 (39%) | 1,477 (43%) |  |
| >=3.5 |  | 2,662 (39%) | 1,895 (39%) | 767 (38%) |  |
| BMI | 10,242 |  |  |  | <0.001 |
| <25 |  | 3,637 (33%) | 2,813 (37%) | 824 (25%) |  |
| 25-30 |  | 3,126 (31%) | 1,986 (29%) | 1,140 (35%) |  |
| ≥30 |  | 3,479 (35%) | 2,273 (33%) | 1,206 (40%) |  |
| Waistline | 10,242 | 98.01± (16.97) | 95.84± (17.13) | 102.80± (15.58) | <0.001 |
| Hypertation | 10,242 |  |  |  | <0.001 |
| without Hypertation |  | 7,196 (72%) | 5,754 (81%) | 1,442 (51%) |  |
| with Hypertation |  | 3,046 (28%) | 1,318 (19%) | 1,728 (49%) |  |
| Diabetes | 10,242 |  |  |  | <0.001 |
| No |  | 6,690 (67%) | 5,001 (73%) | 1,689 (56%) |  |
| Yes |  | 1,225 (10%) | 468 (5.9%) | 757 (19%) |  |
| Borderline |  | 2,327 (23%) | 1,603 (21%) | 724 (25%) |  |
| Smoking | 10,242 |  |  |  | 0.004 |
| no |  | 7,777 (78%) | 5,406 (79%) | 2,371 (75%) |  |
| yes |  | 2,465 (22%) | 1,666 (21%) | 799 (25%) |  |
| Alcohol consumption | 10,242 |  |  |  | <0.001 |
| no |  | 3,423 (22%) | 2,808 (25%) | 615 (14%) |  |
| yes |  | 6,819 (78%) | 4,264 (75%) | 2,555 (86%) |  |
| Noise at work | 10,242 |  |  |  | <0.001 |
| no |  | 7,197 (68%) | 5,295 (72%) | 1,902 (60%) |  |
| yes |  | 3,045 (32%) | 1,777 (28%) | 1,268 (40%) |  |
| Noise in life | 10,242 |  |  |  | 0.400 |
| no |  | 8,516 (84%) | 5,820 (83%) | 2,696 (84%) |  |
| yes |  | 1,726 (16%) | 1,252 (17%) | 474 (16%) |  |
| BRI | 10,242 | 5.22± (2.38) | 4.93± (2.39) | 5.86± (2.21) | <0.001 |
| BRI group | 10,242 |  |  |  | <0.001 |
| 1 |  | 2,561 (23%) | 2,289 (30%) | 272 (8.2%) |  |
| 2 |  | 2,560 (26%) | 1,770 (26%) | 790 (26%) |  |
| 3 |  | 2,560 (26%) | 1,516 (23%) | 1,044 (33%) |  |
| 4 |  | 2,561 (25%) | 1,497 (22%) | 1,064 (33%) |  |
| ^1^N not Missing (unweighted) | | | | | |
| ^2^n (unweighted) (%); Mean± (SD) | | | | | |
| ^3^Pearson's X^2: Rao & Scott adjustment; Design-based KruskalWallis test | | | | | |

**Supplementary Table 2:** Baseline Characteristics of Participants with Speech-Frequency Hearing Loss

The table details the baseline characteristics of participants based on the presence of speech-frequency hearing loss. Demographic, clinical, and lifestyle factors are included, alongside BRI distributions. Pearson’s chi-square test was applied to evaluate the statistical significance of categorical variables, and the Kruskal-Wallis test was used for continuous variables. Participants with speech-frequency hearing loss were more likely to have elevated BRI, older age, and greater exposure to noise at work. Statistical significance for these trends was confirmed (p < 0.001).

| Variables | Model1 | |  | Model2 | |  | Model3 | |  | Model4 | |
| --- | --- | --- | --- | --- | --- | --- | --- | --- | --- | --- | --- |
|  | OR (95%CI) | *P* |  | OR (95%CI) | *P* |  | OR (95%CI) | *P* |  | OR (95%CI) | *P* |
| BRI | 1.17 (1.14 ~ 1.21) | <.001 |  | 1.53 (1.40 ~ 1.68) | <.001 |  | 1.44 (1.32 ~ 1.57) | <.001 |  | 1.44 (1.32 ~ 1.57) | <.001 |
| BRI group | | | | |  |  |  |  |  |  |  |
| Q1 | 1.00 (Reference) |  |  | 1.00 (Reference) |  |  | 1.00 (Reference) |  |  | 1.00 (Reference) |  |
| Q2 | 3.63 (2.87 ~ 4.59) | <.001 |  | 2.23 (1.75 ~ 2.85) | <.001 |  | 2.17 (1.67 ~ 2.81) | <.001 |  | 2.17 (1.67 ~ 2.83) | <.001 |
| Q3 | 5.25 (4.22 ~ 6.53) | <.001 |  | 3.26 (2.52 ~ 4.22) | <.001 |  | 2.99 (2.28 ~ 3.93) | <.001 |  | 2.98 (2.26 ~ 3.93) | <.001 |
| Q4 | 5.35 (4.27 ~ 6.70) | <.001 |  | 5.76 (4.02 ~ 8.26) | <.001 |  | 4.85 (3.31 ~ 7.10) | <.001 |  | 4.85 (3.31 ~ 7.11) | <.001 |
| P for trend | <.001 | |  | <.001 | |  | <.001 | |  | <.001 | |
| OR: Odds Ratio, CI: Confidence Interval | | | | | | | | | | | |
| Model1: Crude | | | | | | | | | | | |
| Model2: Adjust: Sex, Age, Race, Education, Income-poverty ration, BMI | | | | | | | | | | | |
| Model3: Adjust: Sex, Age, Race, Education, Income-poverty ration, BMI, Waistline, Alcohol consumption, Smoking, Hypertension, Diabetes | | | | | | | | | | | |
| Model4: Adjust: Sex, Age, Race, Education, Income-poverty ration, BMI, Waistline, Alcohol consumption, Smoking, Hypertension, Diabetes, Noise at work, Noise in life | | | | | | | | | | | |

**Supplementary Table 3**. Association Between BRI and High-Frequency Hearing Loss

| Variables | Model1 | |  | Model2 | |  | Model3 | |  | Model4 | |
| --- | --- | --- | --- | --- | --- | --- | --- | --- | --- | --- | --- |
|  | OR (95%CI) | *P* |  | OR (95%CI) | *P* |  | OR (95%CI) | *P* |  | OR (95%CI) | *P* |
| BRI | 1.12 (1.10 ~ 1.15) | <.001 |  | 1.52 (1.32 ~ 1.75) | <.001 |  | 1.46 (1.26 ~ 1.68) | <.001 |  | 1.46 (1.27 ~ 1.68) | <.001 |
| BRI group | | | | |  |  |  |  |  |  |  |
| Q1 | 1.00 (Reference) |  |  | 1.00 (Reference) |  |  | 1.00 (Reference) |  |  | 1.00 (Reference) |  |
| Q2 | 3.22 (2.39 ~ 4.35) | <.001 |  | 1.91 (1.33 ~ 2.76) | <.001 |  | 1.88 (1.31 ~ 2.71) | <.001 |  | 1.89 (1.31 ~ 2.73) | <.001 |
| Q3 | 4.53 (3.28 ~ 6.25) | <.001 |  | 2.92 (1.91 ~ 4.47) | <.001 |  | 2.77 (1.83 ~ 4.20) | <.001 |  | 2.78 (1.83 ~ 4.22) | <.001 |
| Q4 | 4.71 (3.60 ~ 6.16) | <.001 |  | 5.51 (3.24 ~ 19.38) | <.001 |  | 4.96 (2.94 ~ 8.36) | <.001 |  | 5.00 (2.96 ~ 8.43) | <.001 |
| P for trend | <.001 | |  | <.001 | |  | <.001 | |  | <.001 | |
| OR: Odds Ratio, CI: Confidence Interval | | | | | | | | | | | |
| Model1: Crude | | | | | | | | | | | |
| Model2: Adjust: Sex, Age, Race, Education, Income-poverty ration, BMI | | | | | | | | | | | |
| Model3: Adjust: Sex, Age, Race, Education, Income-poverty ration, BMI, Waistline, Alcohol consumption, Smoking, Hypertension, Diabetes | | | | | | | | | | | |
| Model4: Adjust: Sex, Age, Race, Education, Income-poverty ration, BMI, Waistline, Alcohol consumption, Smoking, Hypertension, Diabetes, Noise at work, Noise in life | | | | | | | | | | | |
|  | | | | | | | | | | | |

**Supplementary Table 4**. Association Between BRI and Speech-Frequency Hearing Loss

|  | OR (95%CI) | p-value |
| --- | --- | --- |
| One—line linear regression model | 1.46 (1.27 ~ 1.68) | <.001 |
| Two—piecewise linear regression model | |  |
| Inflection point | 6.23 | |
| ＜6.23 | 2.10 (1.76 ~ 2.27) | <.001 |
| ＞6.23 | 1.46 (1.33 ~ 1.60) | <.001 |
| Log—likelihood ratio test |  | 0.001 |
| Sex, Age, Race, Education, Income-poverty ration, BMI, Waistline, Alcohol consumption, Smoking, Hypertension, Diabetes, Noise at work, Noise in life | | |

**Supplementary Table 5**. Threshold Effect Analysis of BRI and High-Frequency Hearing Loss

|  | OR (95%CI) | p-value |
| --- | --- | --- |
| One—line linear regression model | 1.66 (1.42 ~ 1.93) | <.001 |
| Two—piecewise linear regression model | |  |
| Inflection point | 7.05 | |
| ＜7.05 | 2.02 (1.76 ~ 2.32) | <.001 |
| ＞7.05 | 1.52 (1.36 ~ 1.71) | <.001 |
| Log—likelihood ratio test |  | 0.001 |
| Sex, Age, Race, Education, Income-poverty ration, BMI, Waistline, Alcohol consumption, Smoking, Hypertension, Diabetes, Noise at work, Noise in life | | |

**Supplementary Table 6**. Threshold Effect Analysis of BRI and Speech-Frequency Hearing Loss
