# Supplementary material for: Association between body roundness index and hearing loss in the United States population: A cross-sectional study
Source: Medicine (Baltimore). 2025 Oct 3;104(40):e44401. doi: 10.1097/MD.0000000000044401 (PMC12499831; doi:10.1097/MD.0000000000044401)

**Supplementary Figure S1.** Subgroup analysis of association between BRI and High-

Frequency Hearing Loss.


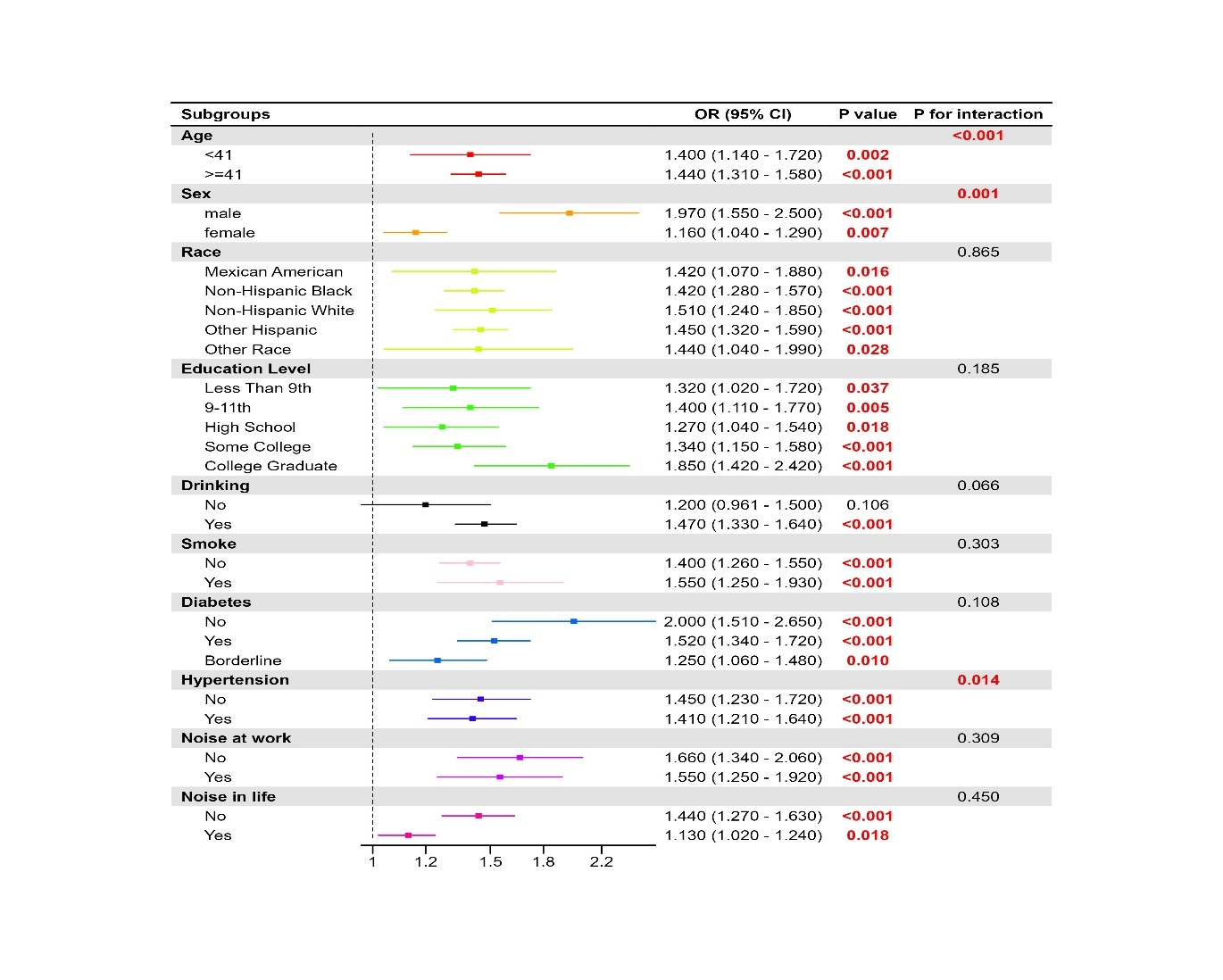

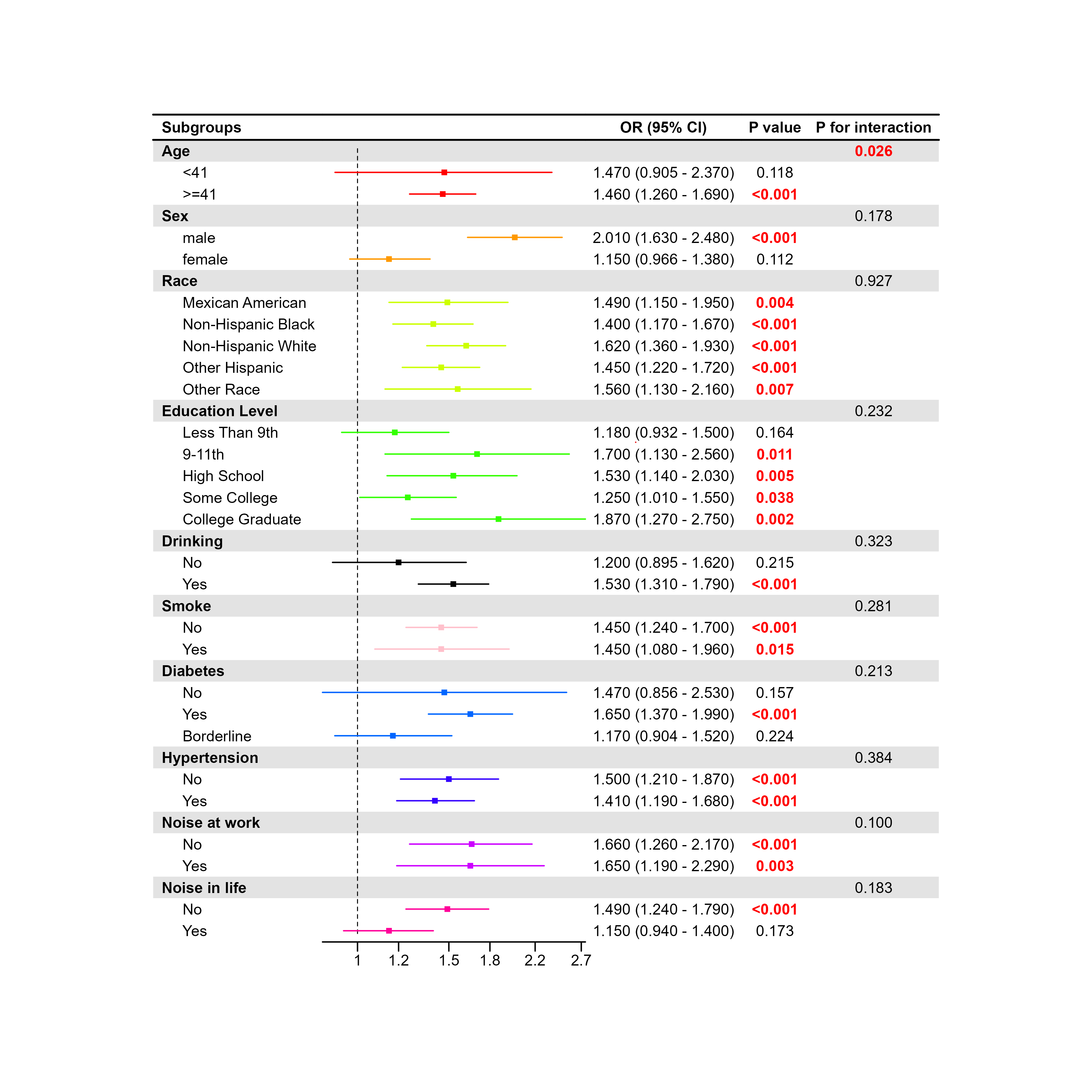


**Supplementary Figure S2.** Subgroup analysis of association between BRI and Speech-Frequency Hearing Loss.


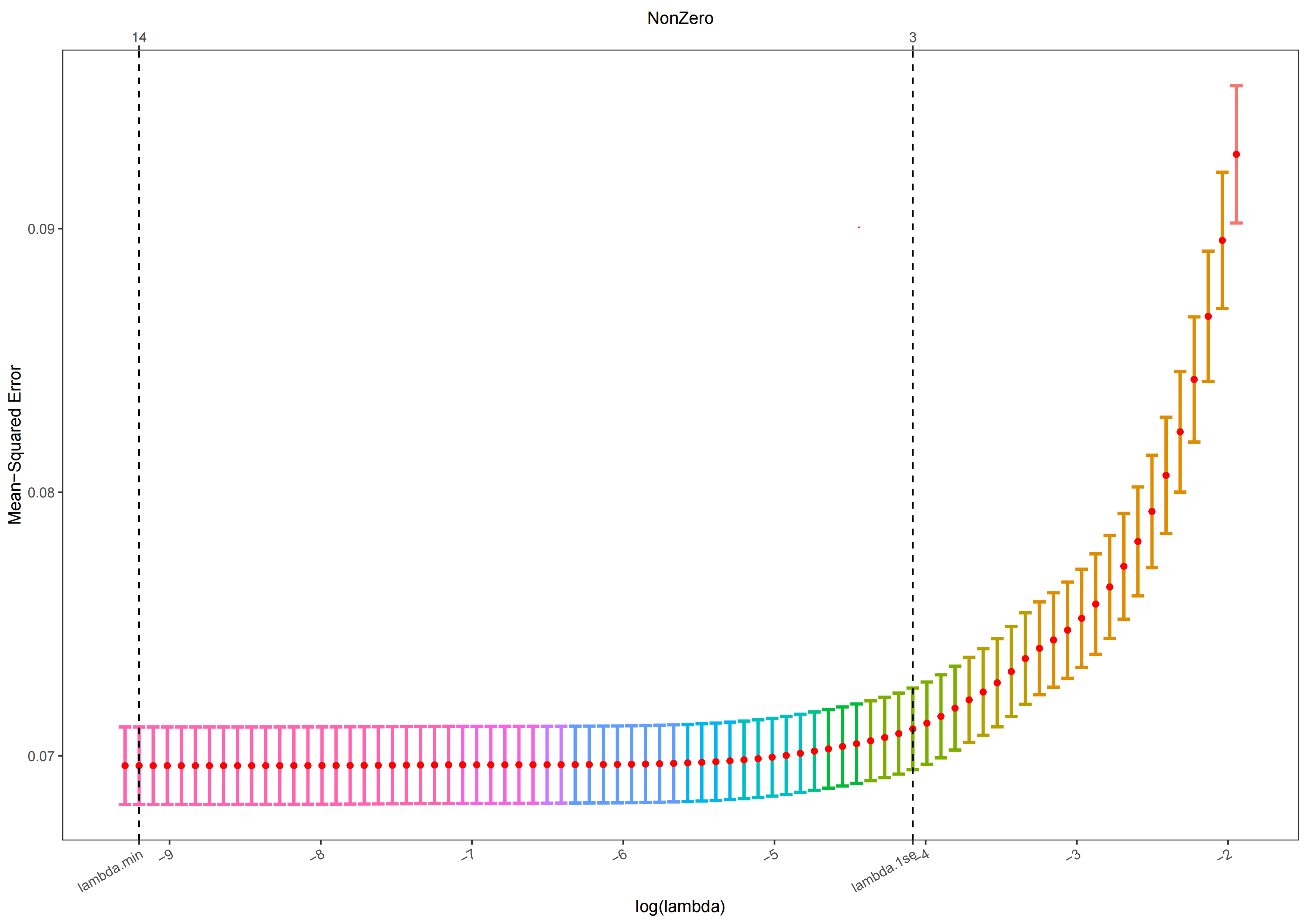


**Supplementary Figure S3.** LASSO Cross-Validation for Low-Frequency Hearing Loss


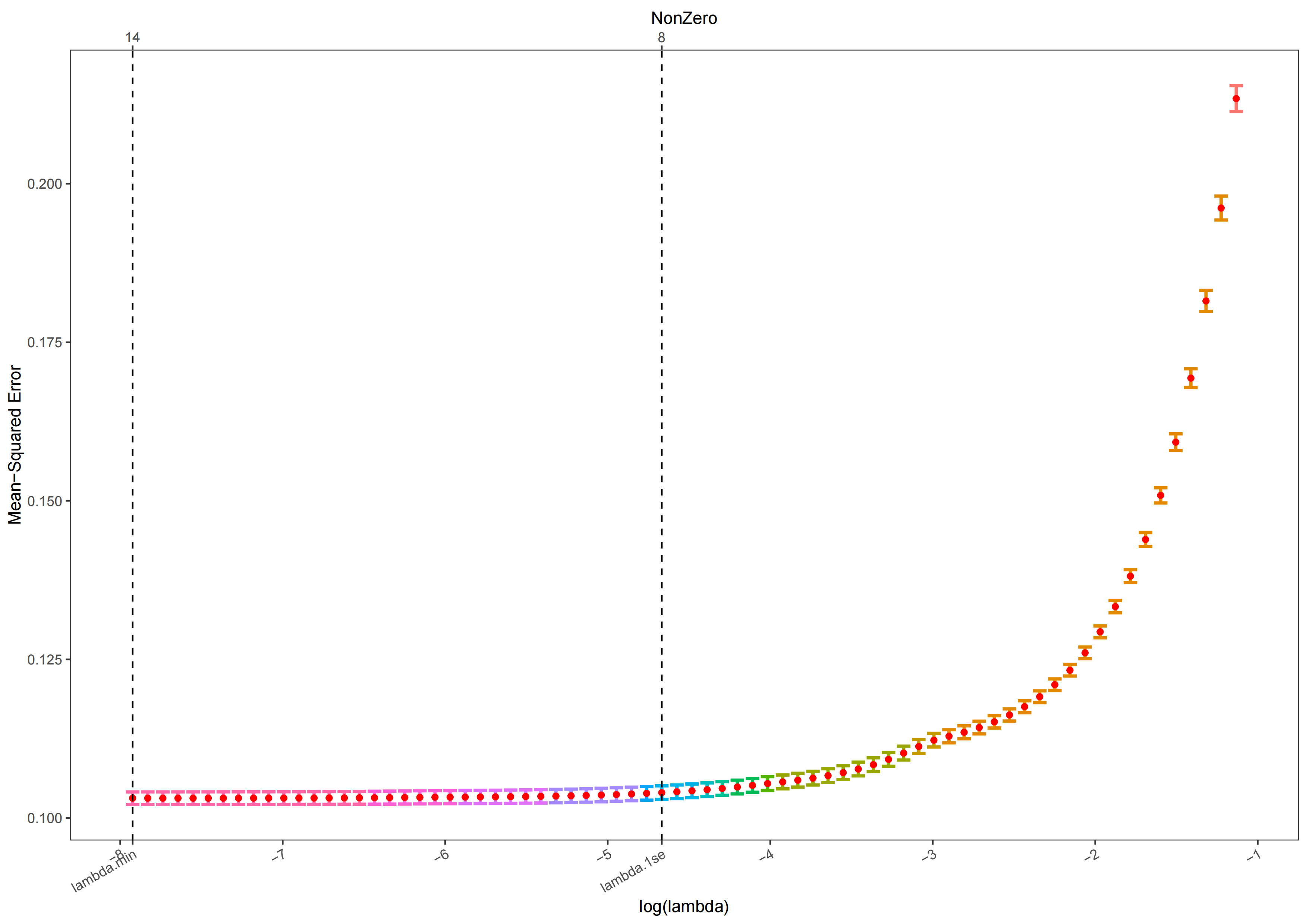


**Supplementary Figure S4.** LASSO Cross-Validation for High-Frequency Hearing Loss


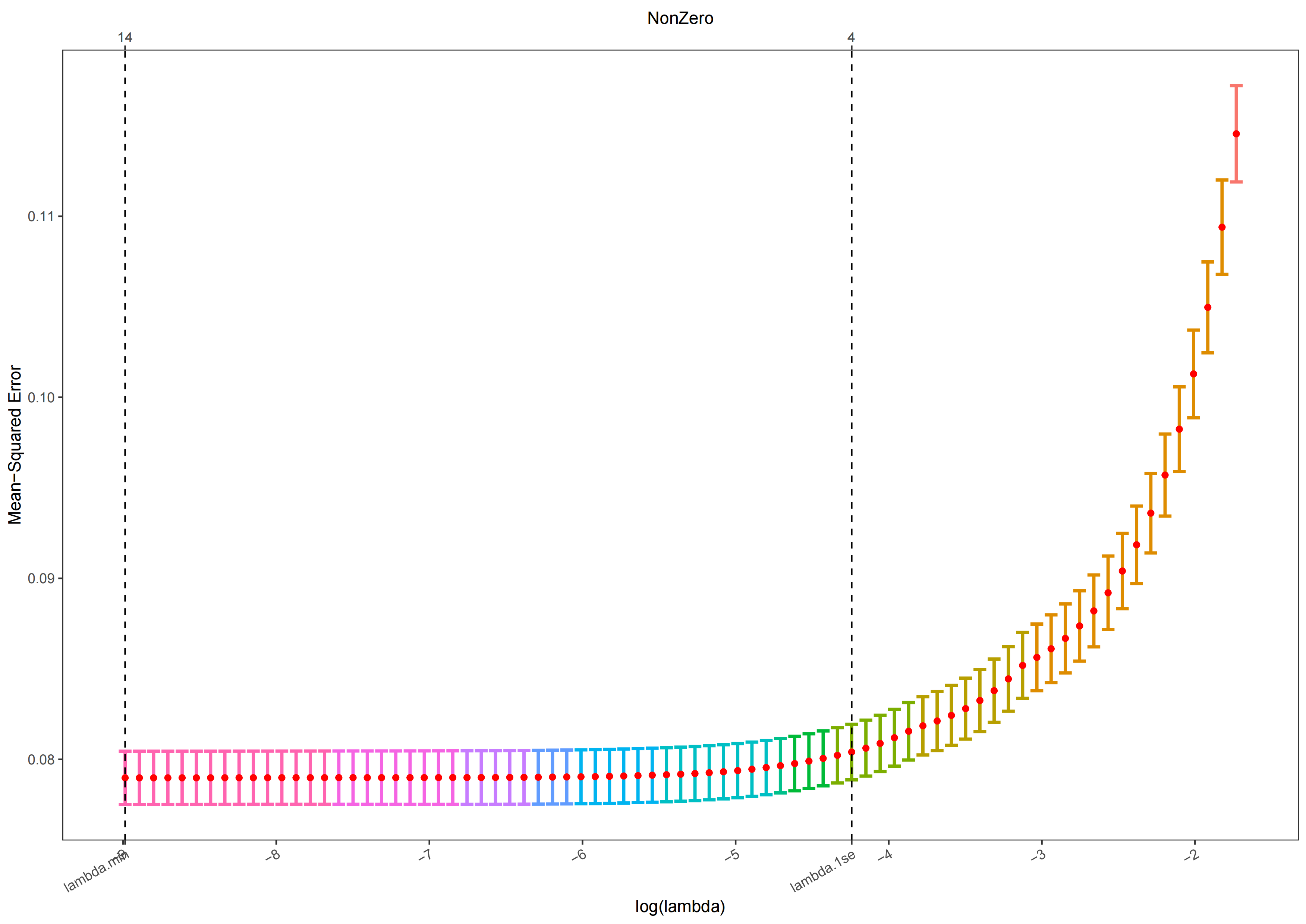


**Supplementary Figure S5.** LASSO Cross-Validation for Speech-Frequency Hearing Loss


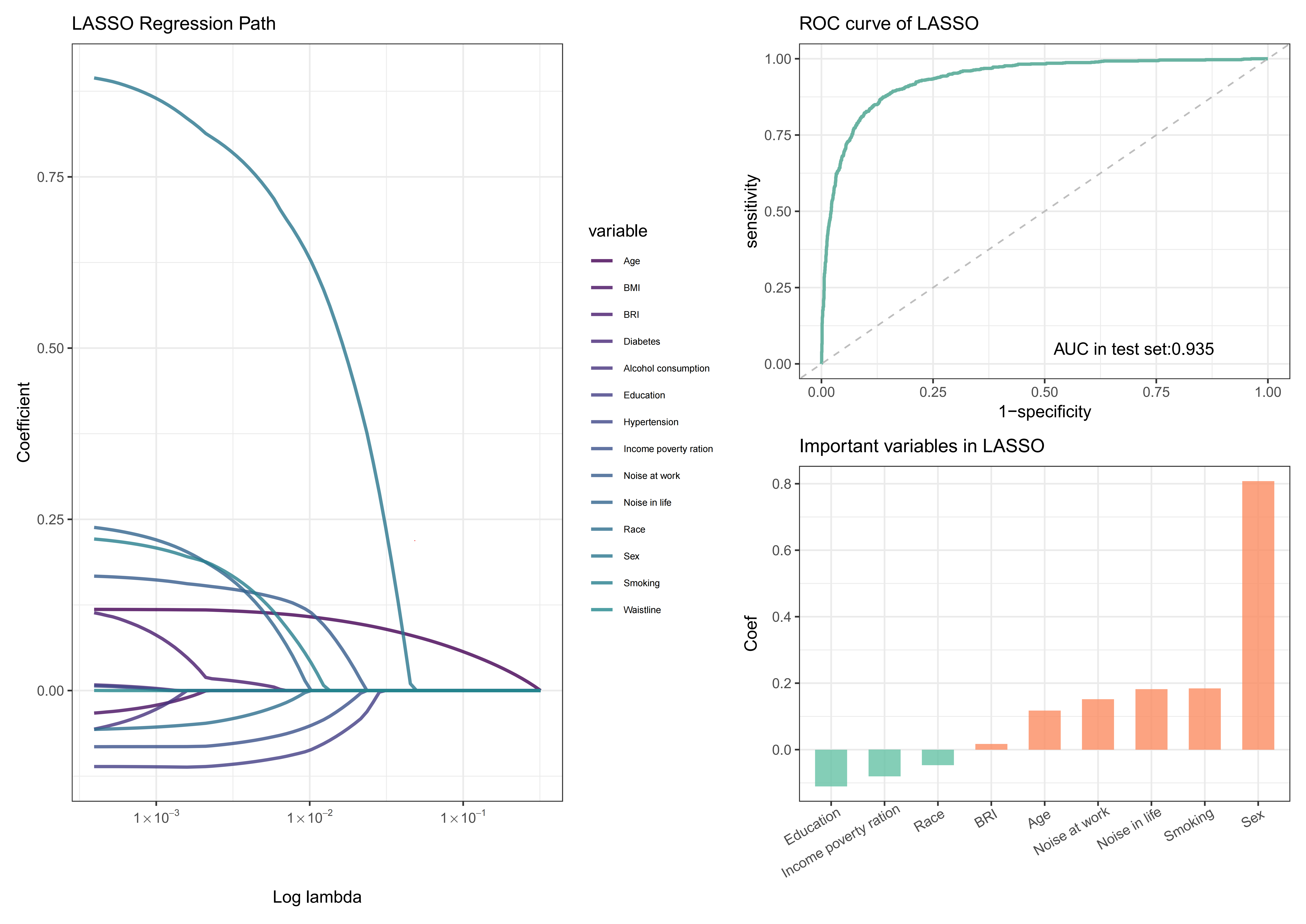


**Supplementary Figure S6.** LASSO Model for High-Frequency Hearing Loss


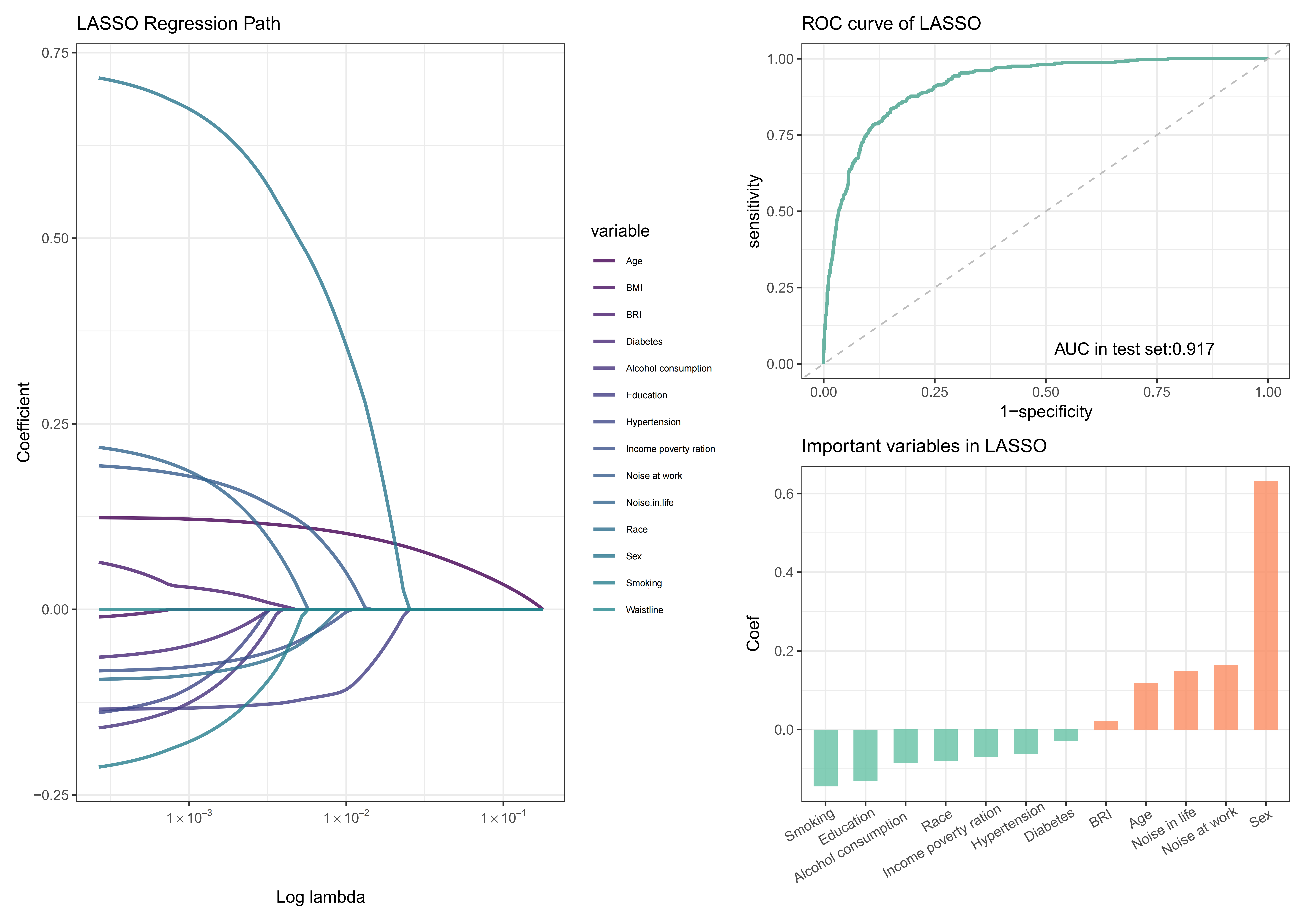


**Supplementary Figure S7.** LASSO Model Feature Selection and Performance for Speech-Frequency Hearing Loss


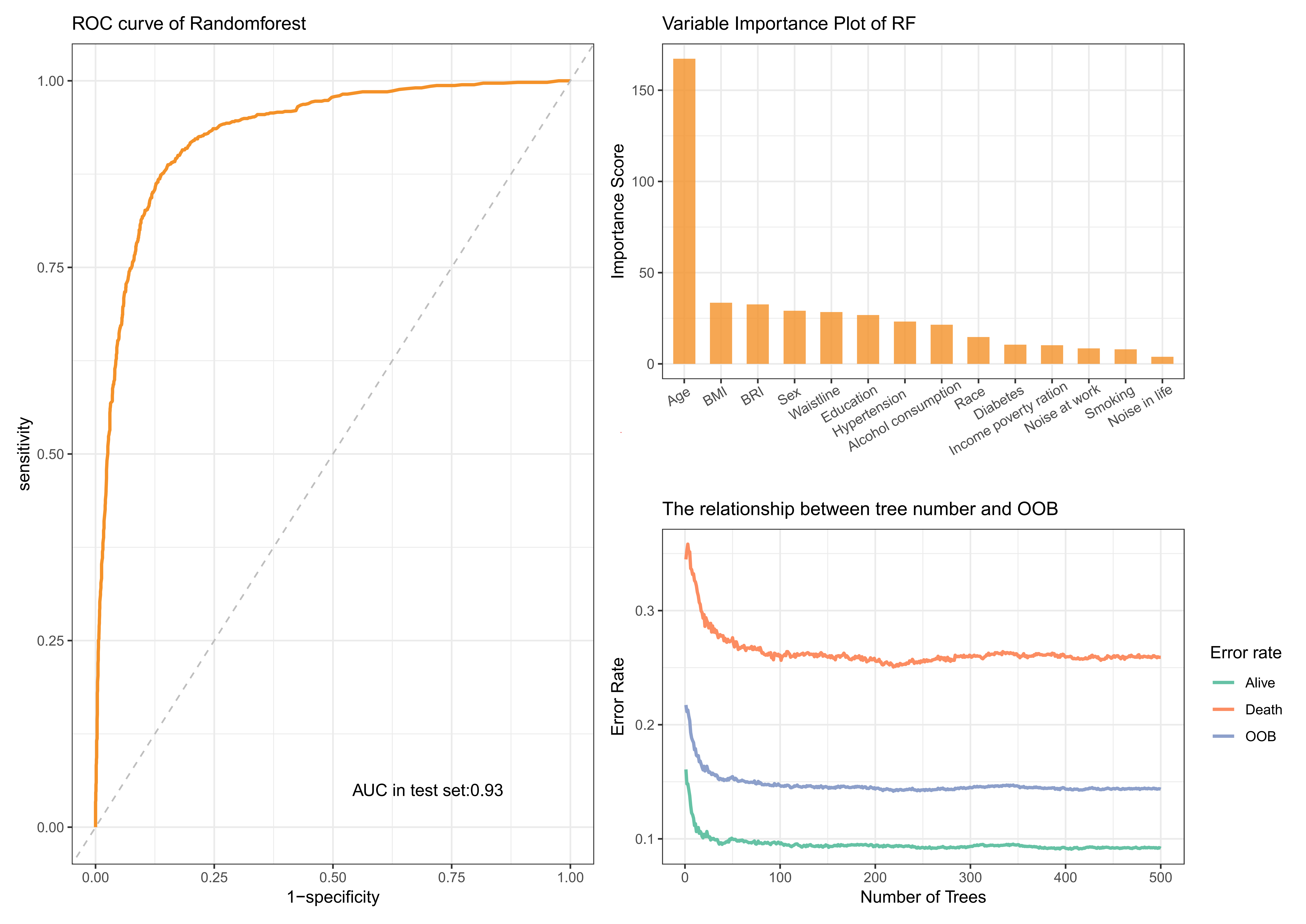


**Supplementary Figure S8.** Random Forest Model Performance and Feature Importance for High-Frequency Hearing Loss


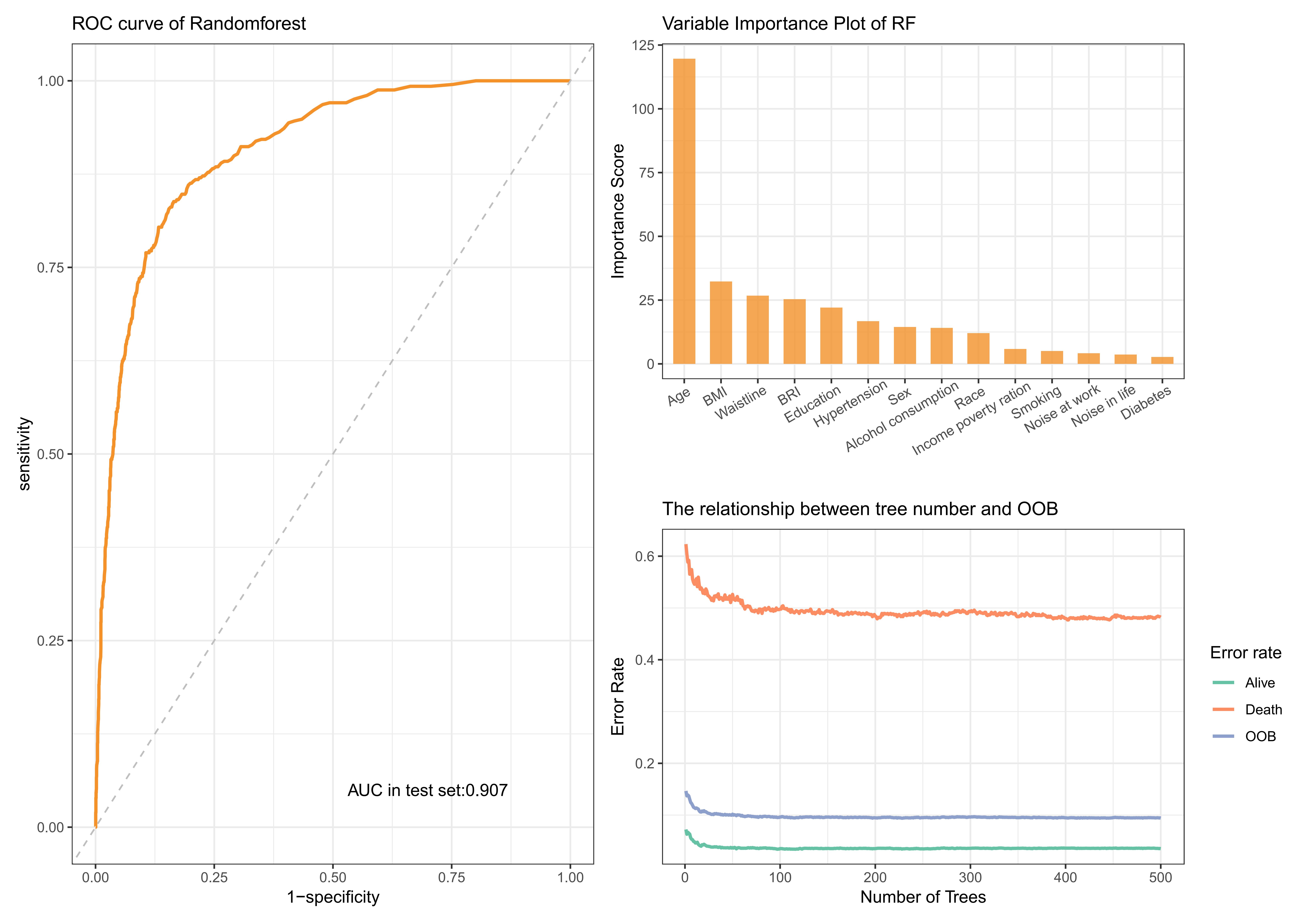


**Supplementary Figure S9.** Random Forest Model Performance and Feature Importance for Speech-Frequency Hearing Loss


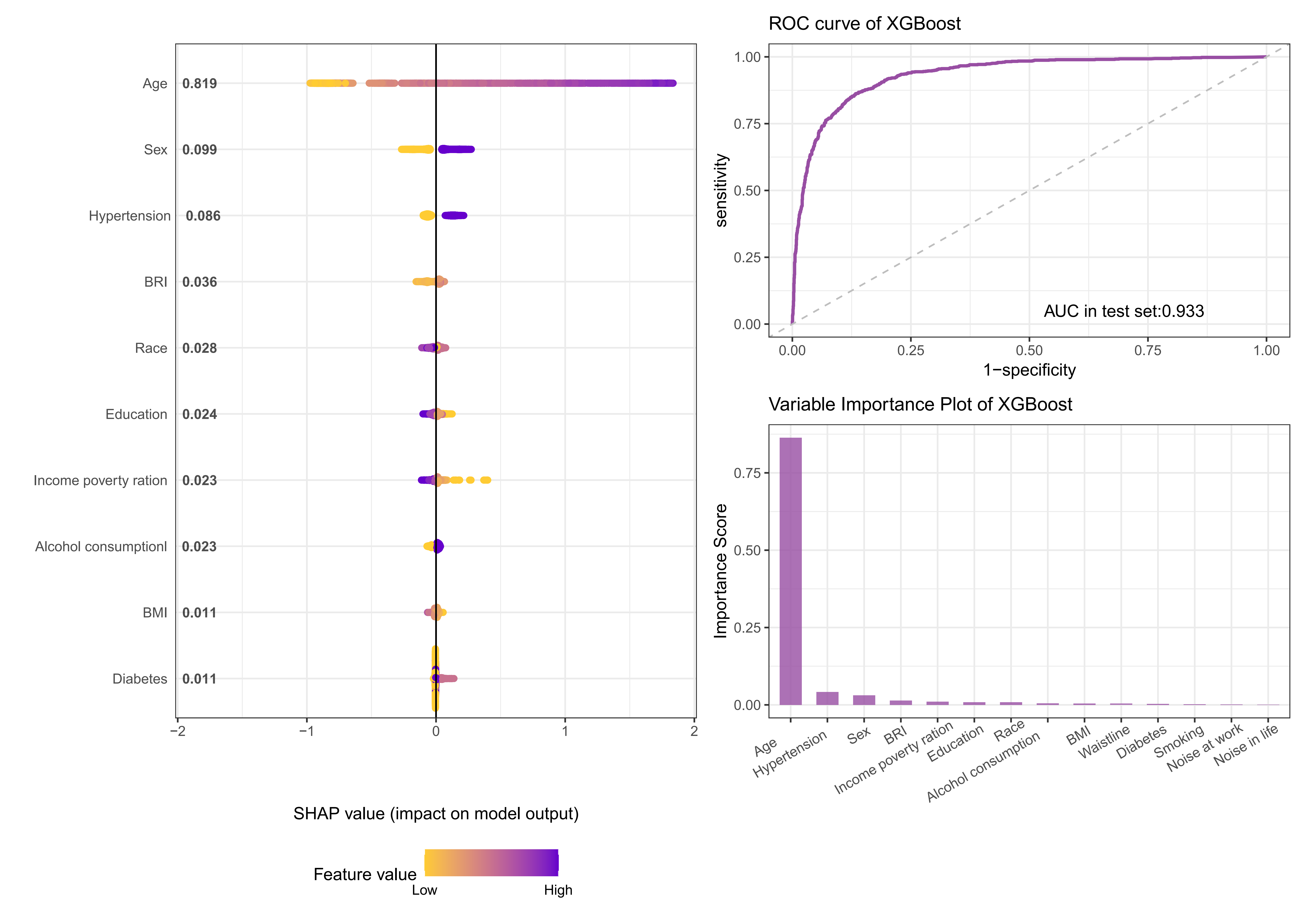


**Supplementary Figure S10.** XGBoost Model Performance and Feature Importance for High-Frequency Hearing Loss

**Supplementary Figure S11.** XGBoost Model Performance and Feature Importance for Speech-Frequency Hearing Loss


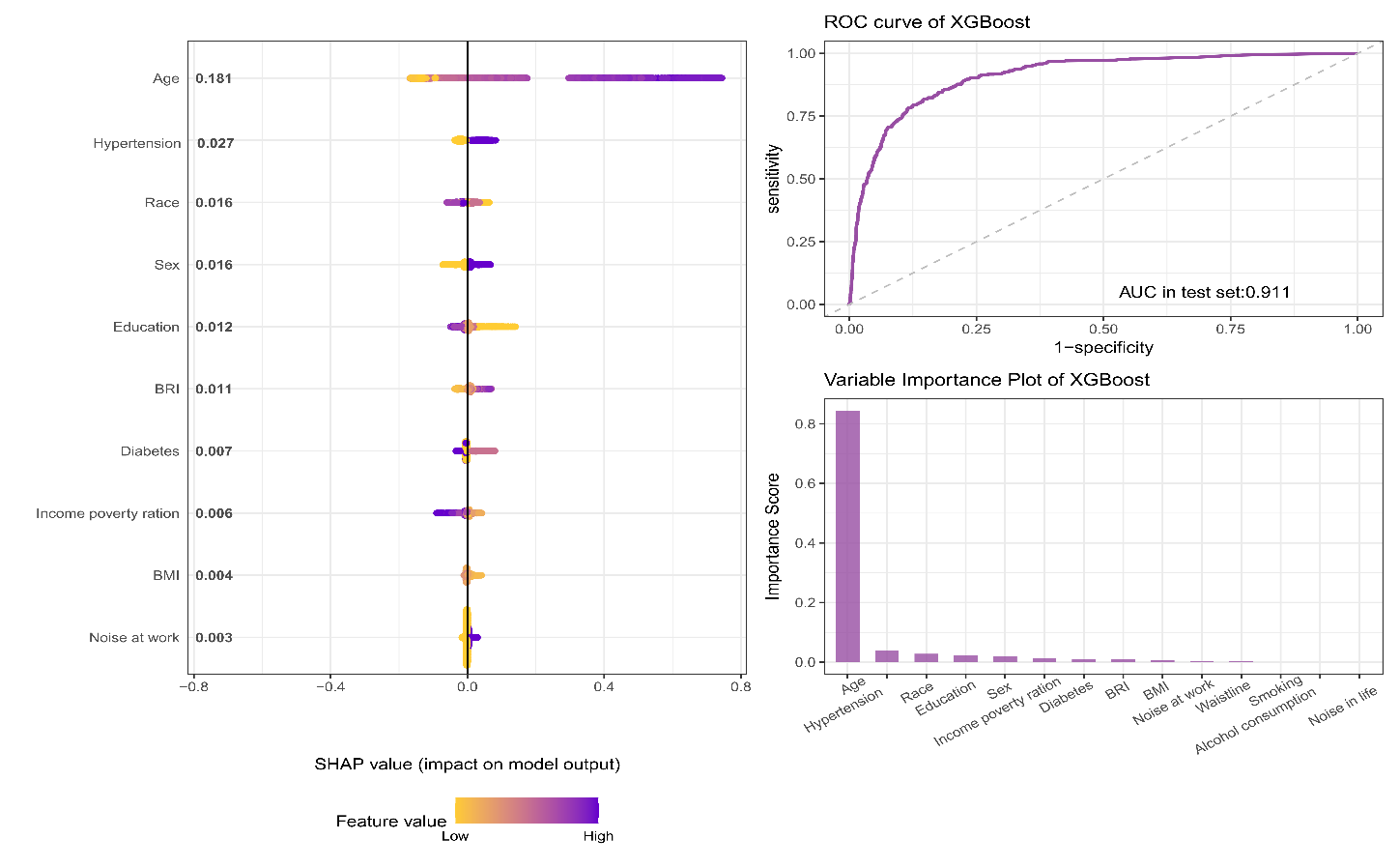

Supplement: Supplementary file 2 [file medi-104-e44401-s002.docx]
